# Accuracy of a screening tool for medication adherence: A systematic review and meta-analysis of the Morisky Medication Adherence Scale-8

**DOI:** 10.1371/journal.pone.0187139

**Published:** 2017-11-02

**Authors:** Sun Jae Moon, Weon-Young Lee, Jin Seub Hwang, Yeon Pyo Hong, Donald E. Morisky

**Affiliations:** 1 Department of Preventive Medicine, College of Medicine, Chung-Ang University, Seoul, Republic of Korea; 2 Department of Computer science and Statistics, Daegu University, Gyeongsan-si, Gyeongsangbuk-do, Republic of Korea; 3 Department of Community Health Sciences UCLA Fielding School of Public Health, Los Angeles, California, United States of America; Universita degli Studi di Perugia, ITALY

## Abstract

**Background:**

This systematic review examined the reliability and validity of the Morisky Medication Adherence Scale-8 (MMAS-8), which has been widely used to assess patient medication adherence in clinical research and medical practice.

**Methods:**

Of 418 studies identified through searching 4 electronic databases, we finally analyzed 28 studies meeting the selection criteria of this study regarding the reliability and validity of MMAS-8 including sensitivity and specificity. Meta-analysis for Cronbach’s α, intraclass correlation coefficient (ICC), sensitivity and specificity to detect a patient with nonadherence to medication were performed. The pooled estimates for Cronbach’s α and ICC were calculated using the random-effects weighted T transformation. A bivariate random-effects model was used to estimate pooled sensitivity and specificity.

**Findings:**

The pooled Cronbach's α estimate for type 2 diabetes group in 7 studies and osteoporosis group in 3 studies were 0.67 (95% Confidence Interval(CI), 0.65 to 0.69) and 0.77 (95% CI, 0.72 to 0.83), respectively. With regard to test-retest, the pooled ICC for type 2 diabetes group in 3 studies and osteoporosis group in 2 studies were 0.81 (95% CI, 0.75 to 0.85) and 0.80 (95% CI, 0.74 to 0.85). For a cut-off value of 6, the pooled sensitivity and specificity in 12 studies were 0.43 (95% CI, 0.33 to 0.53) and 0.73 (95% CI, 0.68 to 0.78), respectively.

**Conclusions:**

The MMAS-8 had acceptable internal consistency and reproducibility in a few diseases like type 2 diabetes. Using the cut-off value of 6, criterion validity was not enough good to validly screen a patient with nonadherence to medication. However, this study did not calculated a pooled estimate for criterion validity using the higher values than 6 as a cut-off value since most of included individual studies did not report criterion validity based on those values.

## Background

Patients with chronic diseases manifesting silent or no symptoms tend to have poor medication adherence [1, 2]. Patient adherence to medication can plays a key role in controlling chronic diseases, since they require continuous treatment for a long time. In this regard, the disadvantaged people with chronic diseases are especially vulnerable to poor adherence to medication due to lack of resources [3, 4]. 40% of all elderly patients with prescriptions do not take their medication as prescribed [5]. According to an EU report, non-adherence to therapies is responsible for 194,500 deaths and costs €125 billion annually [6]. Despite the significant implications of medication nonadherence in a society, many clinicians are not properly trained to screen for non-adherence and instead rely solely on their own empirical judgment [7]. Since medication adherence is a complicated multifactorial behavior, it is important to ensure the accurate and practical tool for measuring medication adherence in routine medical practice to understand the medication behavior of patient [8].

The methods assessing medication adherence can be classified into direct and indirect methods of measurement. Directly observed therapy, measurement of concentrations of a drug or its metabolite in the blood or urine are instances of direct methods to measure adherence to medication. Indirect methods include patients’ questionnaire, pill counts, rates of prescription fills, assessment of the patient’s clinical outcome and electric medication monitors. Each method has strengths and weakness and one of them could be a reference standard for another method [9, 10]. Patient questionnaire is widely used to measure adherence to therapies in the clinical setting due to its simplicity and low cost, even though it is subject to bias of results by patients [11]. One of the most frequently used patient questionnaires for the assessment of medication adherence is the Morisky Medication Adherence Scale (MMAS) [8].

Morisky et al. developed a self-reported scale with 4 items regarding common medication-taking behaviours leading to omission of drug [12]. It had previously been widely used, especially in Randomized Controlled Trials (RCT) of medication adherence intervention among patients with numerous chronic diseases [13]. Later, additional 4 items addressing the circumstances surrounding adherence behavior were supplemented to the original version to overcome some of its limitations; this updated scale was named the 8-item Morisky Medication Adherence Scale (MMAS-8). The MMAS-8 consists of 8 items, the first 7 of which are yes/no questions, and the last of which is a 5 point Likert-scale rating [14]. Since its development in 2009 until the present, the MMAS-8 has been used in more than 200 studies. Over the past 2–3 years, the use of the MMAS-8 in RCTs of medical adherence intervention regarding numerous chronic diseases has increased dramatically. Specifically, the MMAS-8 has been used in 12 RCTs investigating acute coronary syndrome [15, 16], diabetes mellitus [17–21], hypertension [22, 23], chronic heart failure [24, 25], and malignant neoplasm [26]. However, to the best of our knowledge, no systematic review has yet investigated the psychometric properties of the MMAS-8 [27]. This study aimed to estimate the summary values of Cronbach’s alpha and interclass correlation (ICC) indicating reliability of diagnostic test, and sensitivity, specificity and diagnostic odds ratio (DOR) as indicators of criterion validity using meta-analysis of the relevant studies among patients with chronic diseases. Additionally, construct validity, known-group validity and convergent validity were examined in the systematic review of the relevant diagnostic studies.

## Method

We developed and followed a protocol for systematic review and meta-analysis of diagnostic studies based on the PRISMA guidelines [28]. We autonomously made a protocol which included instructions for subject retrieval, database selection, search strategy, inclusion and exclusion criteria, quality assessment, and merging of results. The PRISMA guideline checklist is given in S1 Appendix and protocol is attached in S2 Appendix.

### Search strategy

Medline, Embase, The Cumulative Index to Nursing and Allied Health Literature (CINAHL), and PsycINFO databases were selected. Pilot searches were conducted from September 2014 to April 2, 2015; the full-scale literature search was conducted on January 14, 2016. The only search filter was publication date. Keywords included Morisky medication adherence scale and a combination of MMAS, 8, and item (s). The overall search strategy was designed to maximize sensitivity. The specific search strings are listed in S3 Appendix.

### Eligibility criteria and selection process

Since the MMAS-8 was first developed in 2008, only literature published on or after this date was included. We retrieved literature published from January 1, 2008 to December 31, 2015 without restriction on publication language. The specific inclusion criteria were as follows: 1) focused on the 8-Item Morisky Medication Adherence Scale (MMAS-8); 2) tested the reliability and validity of the MMAS-8; and 3) presented the sensitivity and specificity of the MMAS-8 at least, even if the reliability was not tested. The reason why this criterion was included is that sensitivity and specificity have been regarded as the most informative indicators of diagnostic accuracy [29], and 4) used direct and indirect methods (e.g. rates of prescription refills, patient’s clinical response, electronic medication monitors and measurement of the biologic marker or metabolite) excluding patient’s questionnaire as a reference standard [11]. The exclusion criteria were as follows: tested the validity of another measurement tool for medication adherence by comparing it to that of the MMAS-8.

The search results from each database were initially screened by EndNote (version X7, Thomson Reuters, New York, NY). Manual cross-check (title, authors, and publication year) was then made to remove all duplicate studies. Based on titles and abstracts, the studies were then screened by 2 reviewer (SJM and WYL) to identify potentially relevant studies according to the inclusion criteria independently. The full texts of these primary hits were then examined by the same reviewers for potential eligibility based on the inclusion and exclusion criteria. In instance of uncertain eligibility, one additional reviewer (YPH) involved in the case, and they reached consensus on eligibility.

### Data extraction and quality assessment

One reviewer (SJM) extracted data from a total of 28 articles. After the initial extraction, a second reviewer (WYL) examined the agreement between the numbers and/or text in the extracted data and those from the original articles. In the case of disagreement between them, the relevant data in the article was re-examined, and the error was corrected.

The following information was collected: authors, year of publication, and country of origin, sample population size, characteristics of the sample subjects (i.e., clinical disease, age, and sex), reference tests, and other variables. Those information was presented in S4 Appendix. The reliability and validity values presented by the authors were also extracted. Cronbach’s α coefficient was chosen as the index of internal consistency; test-retest reliability was assessed by intraclass correlation coefficient (ICC) and Spearman’s rank correlation coefficient. The validity indices used included sensitivity, specificity, positive predictive value, negative predictive value for criterion validity. Data from each study was also retrieved, and the sensitivity and specificity were manually calculated from a 2 by 2 table. The calculated values were compared with those in the text. One of studies included had disagreement between sensitivity and specificity calculated from tables and those described in the text from the same original article. We tried to contact the corresponding author of the article to make questions about this discrepancy, but it failed. We selected those values in the table rather than in the text considering the number of study subjects and figures in other tables. The specific data are listed in S6 Appendix.

Study quality was independently assessed by 2 reviewer (SJM, WYL), using the Quality Assessment of Diagnostic Accuracy Studies Criteria—2 (QUADAS-2) scale and its detailed guidelines [30]. The QUADAS-2 assessment comprises 4 domains: patient selection, index test, reference standard, and flow and timing. Each domain is assessed in terms of risk of bias; the first 3 domains are also assessed in terms of concerns regarding applicability [31]. In patient selection domain, risk of bias was examined in the process of selection and exclusion of patients as study subjects. Index test and reference standard domains made the assessment of introduction of risk of bias in their implementation and interpretation. Flow and timing domain reviewed the time interval between index test and reference standard test and evaluated whether all of study subjects got the common reference standards test. [31]. The evaluation of a reference standard (domain 3) was made only in studies presenting criterion validity.

### Evidence synthesis

Meta-analysis was performed for the reliability indices [(Cronbach's α coefficient and ICC)] and the criterion validity indices (sensitivity, specificity and DOR). Other types of evidence including known group validity, convergent validity, and construct validity were synthesized by qualitative method. The distributions of these values are presented based on individual studies; 95% confidence intervals were used in the meta-analysis to estimate the population parameter for sensitivity, specificity, Cronbach's α coefficient, and ICC values. To perform meta-analysis of pooled Cronbach’s α coefficients and ICC using random effects model, all coefficients were transformed to Fisher’s Z values and weighted by sample size (inverse variance weight for the analysis. Inversely transformed values were then used to calculate the pooled estimate and confidence interval for each index [32].

The true positive (TP) or sensitivity (medication non-adherence detected by a reference standard with a MMAS-8 score < 6), true negative (TN) or specificity (medication adherence detected by a reference standard with a MMAS-8 score ≥ 6), false positive (FP), and false negative (FN) values of each study were calculated using MMAS-8 cut-off scores of 6 (S6 Appendix). This threshold was suggested by Morisky, developer of MMAS-8, as a criterion of good adherence to medication [14]. Additionally, diagnostic odds ratio (DOR) in each study as a single measure of diagnostic accuracy was computed with their 95% confidence intervals using its sensitivity and specificity, and we synthesized the pooled diagnostic odds ratio (DOR) value under the random effect model by weighting the respective inverse variance weight (S6 Appendix). To synthesize diagnostic accuracy results of included studies, bivariate random effects model was used. Parameter estimates of this model are the summary receiver operating characteristics (SROC) curve, the summary operating point (summary values for sensitivity and specificity), a 95% confidence region around the summary operating point and a 95% prediction region based the cut-off 6 of common threshold [33]. The reasons for selecting bivariate model were for it to be a sort of hierarchical model and to focus on the estimation of the summary sensitivity and specificity points [34]. Hierarchical model in the meta-analysis of diagnostic studies can examine heterogeneity in test accuracy between the included studies. In addition, as the included articles in this study has common positivity threshold of 6, it could contribute to more valid estimation of summary sensitivity and specificity by pooling studies than a meta-analysis with mixed thresholds. The SROC graph is conceptually very similar to the receiver operating characteristic (ROC) except that each data point comes from a different study, not a different threshold [35]. The advantage of SROC is that accuracy is plotted for different thresholds [35]. It can make the estimation of overall test accuracy which is measured by the closeness of the graph to the top left corner representing high sensitivity and specificity. Moreover, this makes the area under curve (AUC), as an single indicator of diagnostic accuracy, which is equal to the probability that if a pair of diseased and non-diseased individuals is selected at random, the diseased individual will be have a higher test result than the non-diseased individual. For example, the random test, allocating positive results half the time, has an AUC of 0.5 [34]. The closer the curve to the unit square indicating AUC equal 1, the better the overall accuracy across different thresholds. Based on the number of false positive, false negative, true positive and true negative of an individual study involved, DOR in each study, a measure of overall test accuracy, was calculated.

To inspect sources of heterogeneity, subgroup analysis using random effects model was undertaken for the Cronbach’s α and ICC instead of multivariable meta-regression, because the number of studies included in this research is insufficient to be put into the multivariable model with comparison to that of variables. First of all, since disease-specific demands for assessment of adherence to medication are useful information for health professionals, disease type was chosen as a variable for subgroup analysis. Afterward, till the disappearance of heterogeneity between individual studies in a subgroup, the subgroup analysis was successively undertaken by other subgroup variables such as characteristics of sample and index test and its measurement as follows: questionnaire language (English vs non-English), number of latent structures of questionnaire (1 vs 2 and over), retest interval (≥4 weeks vs below), proportion of age group (≥60 mean ages below), proportion of female sex (≥50% vs below), percentage of participants with low adherence group (≥6 vs below) and celling effect with percentage of participants with MMAS-8 score of 8 (≥15% vs below). Cohen’s Q statistics and the Chi-square test were used to investigate heterogeneity within the meta-analysis, but p value less than 0.1 on Chi-square test was criteria of judging heterogeneity.

A criteria of judging heterogeneity across diagnostic accuracy studies in bivariate model is whether an individual study is out of a 95% prediction region around summary operating point in SROC graph [34]. Subsequently, subgroup analysis by disease type was undertaken on the bivariate model, since statistical heterogeneity may not necessarily predict clinical heterogeneity as different disease or different reference standard. The shape of SROCs and their AUCs between the results of subgroup analysis by disease type were compared to examine clinical heterogeneity between diseases. Although subgroup reference standard would be preferred to judge clinical heterogeneity than disease type, the number of individual studies per a reference standard was not enough to undertake bivariate model analysis. Moreover subgroup analysis by disease type on random effect model of DOR was undertaken to review heterogeneity between individual studies per subgroup. The “mada” package in R (version 3.1.3) was used to conduct the meta-analysis of criterion validity and R (version 3.1.3) of "metafor" package of meta-analysis of Cronbach’s alpha and ICC.

### Additional analysis

To determine the impact of each individual item of MMAS-8 on reliability and validity, adherent response rates and Cronbach’s α coefficients if deleted were extracted from each study presenting those values.

## Results

### Search and study selection

**Fig 1:**
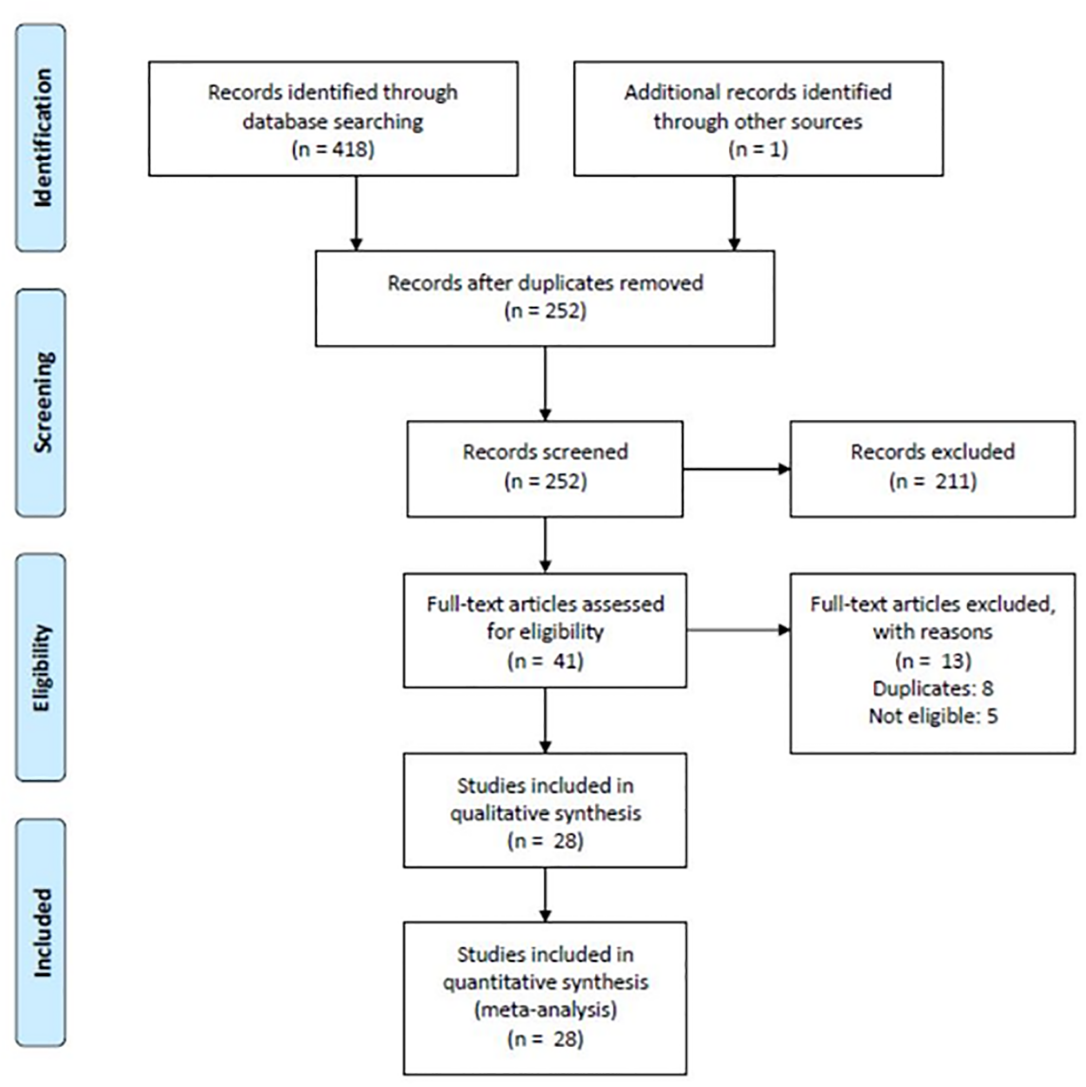
Flow chart of evidence selection.

Our literature search identified 418 papers in the 4 databases. After removing duplicates and performing manual cross-checking, 251 articles remained; one additional article [14] that primarily reported MMAS-8 was included, yielding a total of 252 screened articles. Of these 252, 211 articles were excluded upon application of the exclusion criteria. After retrieving the full-text versions of 41 articles, 13 articles were excluded due to ineligibility(n = 5) overlap (n = 8). We finally analyzed 28 articles, quantitative evidence synthesis for the internal reliability of 25 articles, test-retest reliability of 10 articles, and criterion validity of 15 articles. Other validity indicators such as construct validity, known-group validity and convergent validity were qualitatively synthesized. An overall flow chart of our search results is shown in Fig 1.

### General characteristics of included studies

A total of 28 studies included 11,157 subjects in total; 10 studies focused on hypertension, 9 on type 2 diabetes mellitus (T2DM), 3 on osteoporosis, 2 on thromboembolic disease and 4 on others (Table 1). Of the 28 studies, 20 used the MMAS-8 scale after translation into a language other than English, 13 of which were conducted in Asian countries. Abstracts alone were available for 4 articles whose work was presented at a conference. Out of 28 records, 12 were self-administered survey and 9 have been administered by face-to-face interview. In 12 out of 28 analyzed test-retest reliability. Retest interval for test-retest reliability was ≤4 weeks for 11 studies and >4 weeks for 1. 10 of the 15 studies did against clinical outcomes, 2 studies conducted how well the scale correlated with metabolites of drugs and the rest 3 studies (Table 1). Cronbach’s α coefficient was analyzed in 25 studies, ICC in 10 studies, and sensitivity and specificity in 15 studies. Furthermore, detailed information about characteristics of individual study is detailed information is provided in S4 Appendix.

**Table 1:** General characteristics of 28 studies included for systematic review.

Characteristic	Studies, n (%)
**Disease**	
Hypertension	10 (35.7)
Type 2 diabetes mellitus	9 (32.1)
Osteoporosis	3 (10.1)
Thromboembolic disease	2 (7.1)
Others^a^	4 (14.3)
**Questionnaire language**	
English version	7 (25.0)
Non-English (translated) version	20 (71.4)
Mixed version^b^	1 (3.6)
**Mean age**	
60 years under	13 (46.4)
60 years or over	14 (50.0)
Unknown	1 (3.6)
**Female %**	
50 over	10 (35.7)
50 or under	17 (60.7)
Unknown	1 (3.6)
**Publication type**	
Journal article	24 (85.7)
Conference abstract	4 (14.3)
**Methods of index test**	
Face-to-face interview	9 (32.1)
Self-administered survey	12 (42.9)
Unknown setting	7 (25.0)
**Retest interval for test-retest reliability**	
4 weeks or under	11 (39.3)
4 weeks over	1 (3.6)
N/A	16 (57.1)
**Reference standard**	
Clinical response	10 (35.7)
Biochemical analysis of drugs^c^	2 (7.1)
Others^d^	3 (10.7)
N/A	13 (46.4)

N/A = not applicable

^a^ Other: inflammatory bowel disease (1 study), seizure (1 study), myocardial infarction (1 study), treatment-resistant hypertension (1 study).

^b^ French and Ewe (native Togo language) versions.

^c^ Drug metabolite (6-thioguanine), therapeutic drug monitoring.

^d^ Medication possession ratio (MPR), fasting blood glucose level (FBS) etc.

### Assessment of methodological quality (QUADAS-2)

#### Judgment of risk of bias

**Fig 2:**
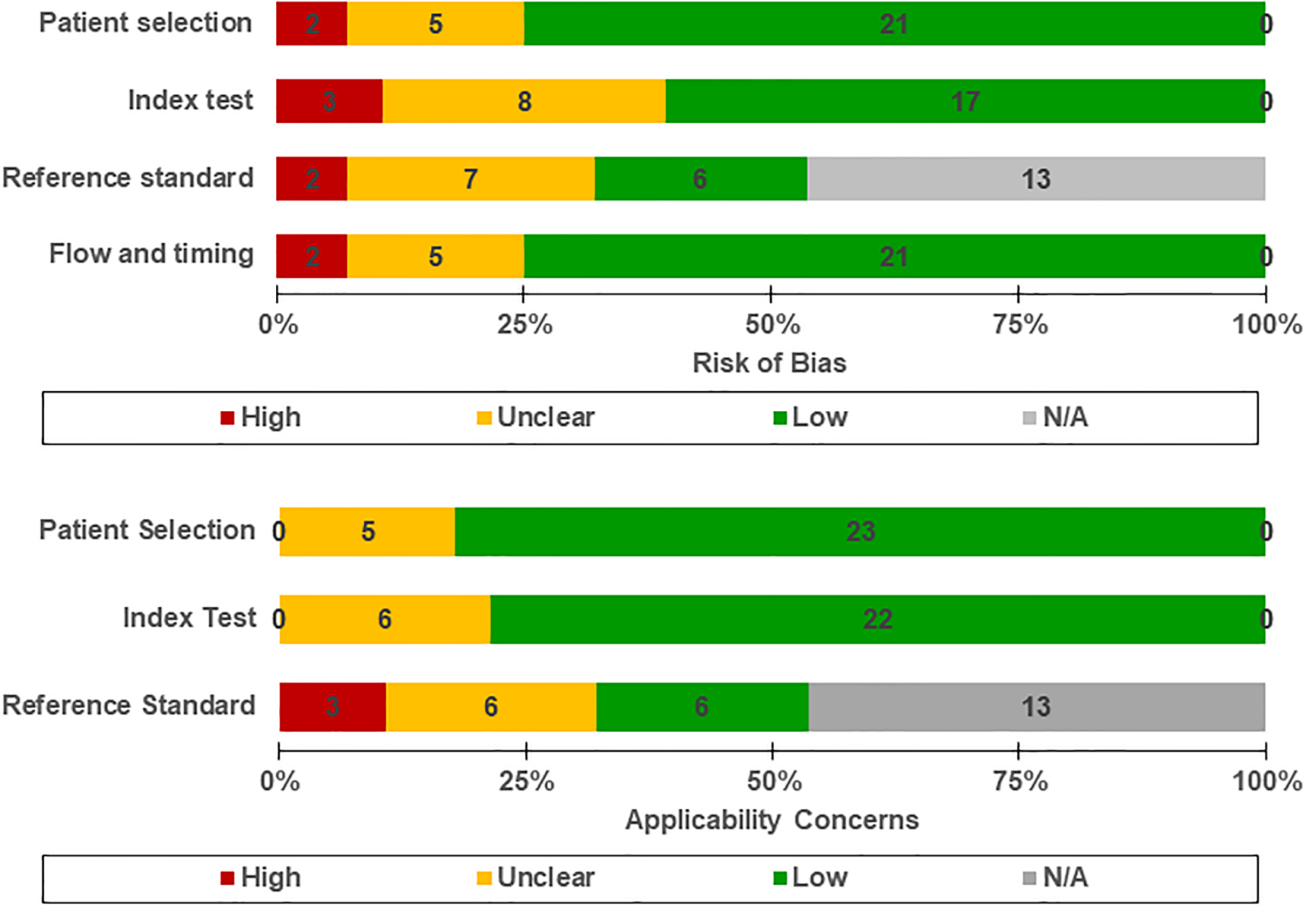
Assessment of methodological quality by QUADAS-2.

Of the 28 studies, low risk of bias with respect to patient selection was detected in 21 studies, unclear risk in 5 studies, and high risk in 2 studies. The 2 studies with high risk of bias exhibited a case-control design and inappropriate exclusion, respectively. For index test, 17 studies had a low risk of bias; 8 and 3 studies had unclear risk and high risk of bias, respectively. Those with high risk of bias had conducted questionnaire surveys with subjects who had been already aware of the reference standard outcome. For the reference standard, we evaluated 15 studies that measured the criterion validity. Reference standard tests in them were presented in S4 Appendix. This assessment revealed 6 studies with a low risk of bias, 7 with unclear risk, and 2 with high risk due to interpretation of the results of the reference test after recognizing the index test results. In terms of flow and timing, 21 studies had a low risk of bias, and 5 had unclear risk. Two studies were regarded as high risk of bias because of discrepancies between the figures described in the patient selection process and the numbers in the tables. The relevant data for these classifications are shown in Fig 2.

#### Judgment of applicability

Of the 28 studies, 23 studies were low risk of bias and 5 were unclear risk of bias with respect to patient selection; no study with high risk of bias wasidentified for this parameter. For index test, 6 studies failed to present situational descriptions when conducting questionnaires and were regarded as unclear risk of bias. Moreover, 15 studies analyzed the or criterion validity in terms of the reference standard; of these, 6 were classified as unclear risk of bias since they did not include a detailed description of a reference standard. Three studies were classified as high risk of bias, one of which used pharmaceutical science students lacking clinical experience to measure blood pressure, and the other of which used reference standards obtained with fasting blood glucose among diabtes patients and electronic blood pressure measuring devices for hypetensive patients, respectively. The relevant data are shown in Fig 2 and S5 Appendix.

### Reliability

#### Internal consistency

Of the 28 studies, 25 used Cronbach's α coefficient to measure internal consistency, 1 used the Kuder-Richardson α coefficient (0.709), and the remaining 2 studies did not use either. The Cronbach's α coefficients ranged between 0.31 and 0.83, a finding that indicates heterogeneity (p < 0.001, I2 = 94.2%).

**Fig 3:**
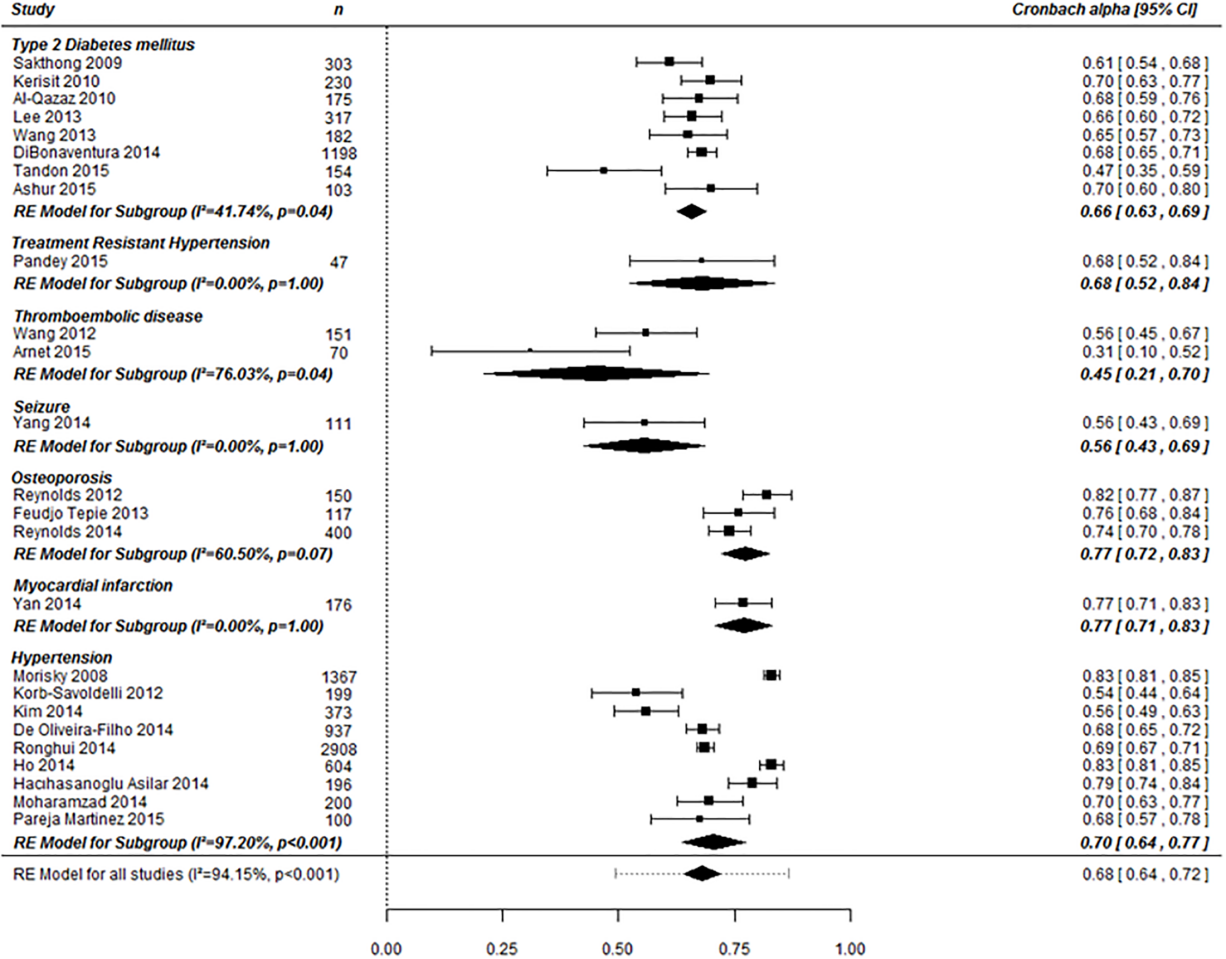
Subgroup analysis of Cronbach's α by disease.

Subgroup analysis by disease revealed that the Cronbach's α coefficients ranged from 0.54–0.83 in the hypertensive group (9 studies), revealing heterogeneity (p < 0.001, I2 = 97.2%), and from 0.47–0.70 in the diabetes group (8 studies), also revealing heterogeneity (p = 0.04, I2 = 41.7%). The 2 thromboembolic disease studies had coefficients of 0.31 and 0.56, also revealing heterogeneity (p = 0.04, I2 = 76.0%). The pooled estimate of the osteoporosis group (3 studies) was 0.77 (95% confidence interval, 0.72 to 0.83); this coefficient revealing heterogeneity (p = 0.07, I2 = 60.5%). The Cronbach's α coefficients of the studies dealing with myocardial infarction, seizure, and treatment–resistant hypertension were 0.77 (0.71 to 0.83), 0.56 (0.43 to 0.69), and 0.68 (0.52 to 0.84), respectively Fig 3.

With respect to the Cronbach's α coefficient, subgroup analysis of the hypertension group resulted in a pooled estimate of 0.58 (95% confidence interval, 0.51 to 0.65) without heterogeneity (p = 0.1847, I2 = 40.8%) in the male-dominant group (percentage of women less than 50%). In contrast, the range was 0.68–083 with heterogeneity (p < 0.0001, I2 = 96.8%) in the female-dominant group (percentage of men less than 50%). We next performed subgroup analysis according to questionnaire language within the female-dominant group with hypertension. After excluding one study using the English version, the pooled estimate of the translated version group (4 studies) was 0.71 (0.67 to 0.74) with heterogeneity (p = 0.0192, I 2 = 69.8%). For further subgroup analysis on those studies with the percentage of participants with low adherence (6 < MMAS score), the pooled estimates of two studies with study subjects with low adherence being less than 50% was 0.69 (0.67 to 0.70) without heterogeneity (p = 0.80, I 2 = 0%), that of the other two studies with study subjects with low adherence being 50 percent and over was 0.75 (0.66 to 0.84) with heterogeneity (p = 0.04, I 2 = 76.3%).

Subgroup analysis of the T2DM group (7 studies) after excluding one study [36] in which Cronbach’s α differed greatly from the others on the forest plot resulted in a pooled estimate of 0.67 (0.65 to 0.69) without heterogeneity (p = 0.5567, *I*^2^ = 0%). The 2 thromboembolic disease studies differed with respect to structure number (4 versus 1), mean age (older than 60 versus younger than 60), language (non-English versus mixed), and ceiling effect with percentages of MMAS-8 score of 8 (64.3% versus 31.1%).

#### Test-retest reliability

Of the 14 studies that examined test-retest reliability, 10 studies calculated the ICC. Spearman’s rank correlation coefficient was calculated in 2 studies, yielding values of 0.82 and 0.93; the weighted kappa statistic was calculated in another 2 studies, yielding values of 0.56 and 0.69; and the Pearson correlation coefficient was calculated in the remaining study, yielding a value of 0.88. The ICC values ranged between 0.24 and 0.91 and were heterogeneous (p < 0.0001, I2 = 88.3%).

**Fig 4:**
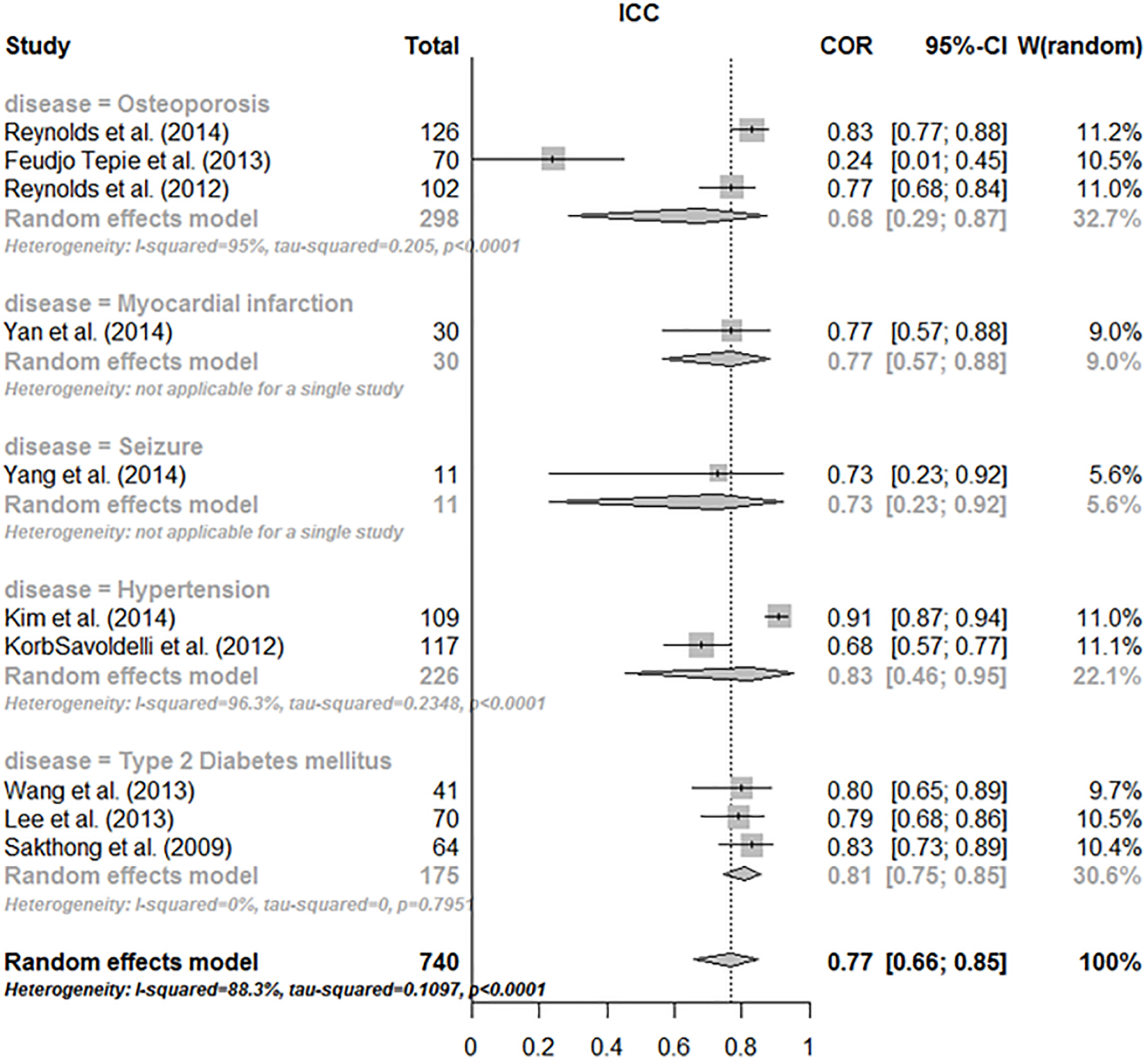
Subgroup analysis of ICC by disease.

Subgroup analysis of the hypertension group (2 studies) resulted in ICC (intraclass correlation) values of 0.68 and 0.91, revealing heterogeneity (p < 0.0001, I2 = 96.3%). The pooled estimate of the T2DM group (3 studies) was 0.81 (95% confidence interval, 0.75 to 0.85) without heterogeneity (p = 0.7951, I2 = 0%), and the range of the osteoporosis group (3 studies) is 0.24–0.83, revealing significant heterogeneity (p = 0.0001, I2 = 88.3%). The diseases not included in the meta-analysis due to having one study in each disease were myocardial infarction, seizure, and thromboembolic disease, which had ICC values of 0.77 (0.57 to 0.88), 0.73 (0.41 to 0.67), and 0.56 (0.44 to 0.66), respectively Fig 4.

With respect to ICC, analysis of the osteoporosis subgroup with less than or equal to 4 weeks of retest-interval (2 studies) resulted in a pooled estimate of 0.80 (95% confidence interval, 0.74 to 0.85) without heterogeneity (p = 0.2139, I2 = 35.3%). The remaining study with a retest-interval greater than 4 weeks (1 study) presented a pooled estimate of 0.24 (0.11 to 0.45). The factors that differed between the 2 hypertension studies were the latent structure number from factor analysis (1 versus 3), the retest-interval (4 or <4 weeks versus > 4 weeks), language (French versus Korean), and low adherence percentage (17.6% versus 32.7%).

### Validity

#### Criterion validity

Criterion validity was measured in 15 studies, of which 14 studies provided criterion validity values by the cut-off value of 6 and the remained one did not provide it. The diseases which the studies dealt with were as follows: hypertension, 6; T2DM, 6; and osteoporosis, and inflammatory bowel disease; 1 each. 5 of 6 studies dealing with hypertension used blood pressure control as a reference standard and the remained one did Medication Possession Ratio (MPR). 5 of 6 studies dealing with T2DM used HbA1c as a reference standard and the rest one did fasting blood sugar. We observed discordance between the sensitivity and specificity values of the text and the values in our 2 by 2 table in one of these studies. We tried to contacted corresponding author of that study but our inquiry did not receive any response. For this case, we used the numbers in our 2 by 2 table.

In an analysis of 14 studies with a cut-off of 6, sensitivity ranged between 0.13 (95% CI 0.06 ~ 0.24) and 0.94 (95% CI 0.74 ~ 0.99), specificity ranged between 0.55 (95% CI 0.46 ~ 0.65) and 0.86 (95% CI 0.76 ~ 0.93) and DORs had the range of 0.67 (95% CI 0.22 ~ 2.33) ~ 30.6 (95% CI 3.94 ~ 237.57) (S6 Appendix).

**Fig 5:**
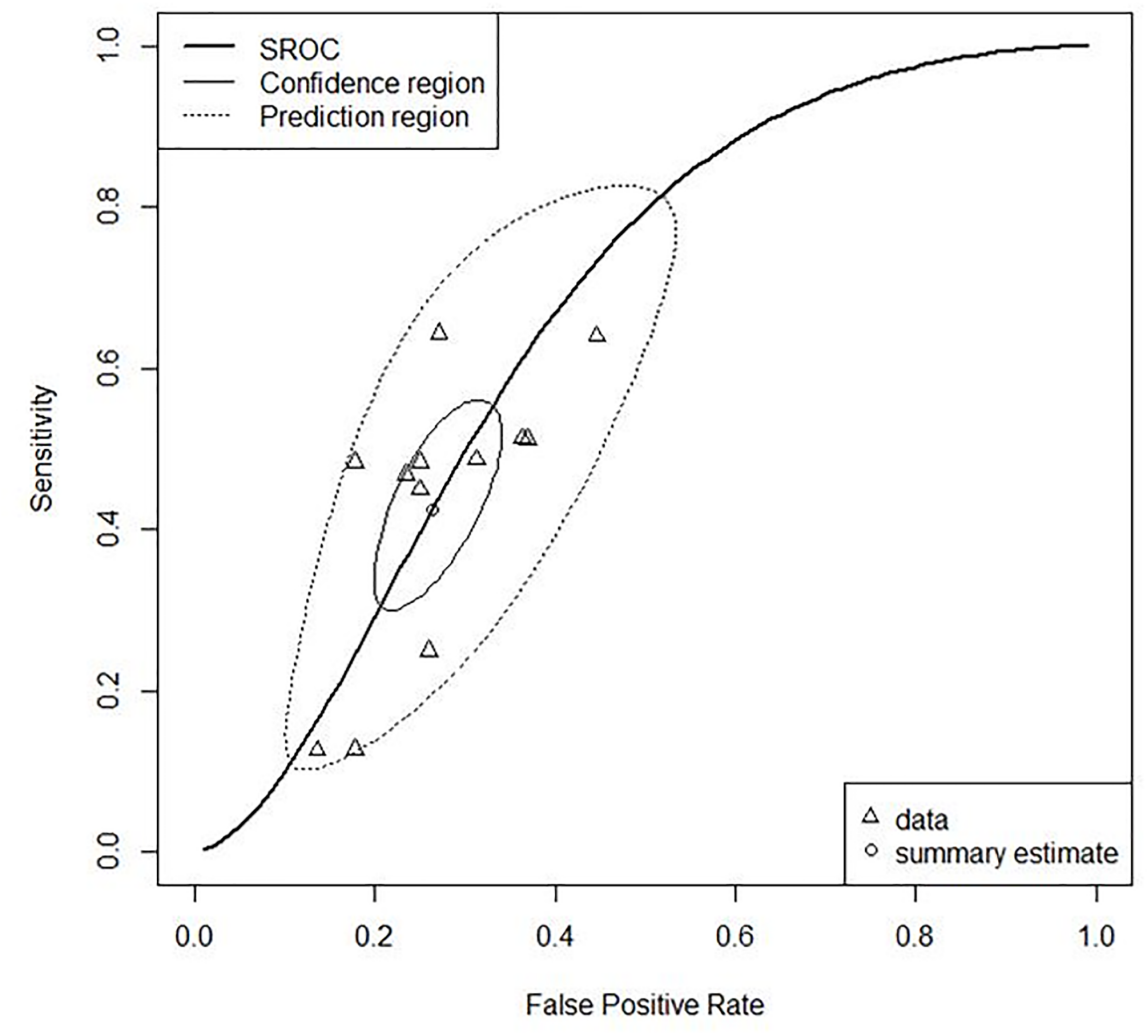
SROC plots of sensitivity and specificity based on cut-off of 6 using bivariate model.

On bivariate model analysis, Goodhand et al’s [37] and Ashur et al’s study [38] were potential outliers since their sensitivity and specificity point on SROC were out of 95% predictive region surrounding summary operating point. Their sensitivity, specificity and DOR had much higher scores than the other studies. It was assumed because their sample had much more disproportionate ratio of good control to poor control of disease than the other studies. Subsequently, except for them, the bivariate model analysis was fitted into data of 12 individual studies using cut-off of 6 as a reference value. The summary point, 95% confidence region, 95% prediction region and SROC curve are shown in Fig 5. Summary sensitivity and specificity are 0.43 (95% CI 0.32 ~ 0.53) and 0.74 (95% CI 0.68 ~ 0.79), respectively. Since any sensitivity and specificity of 12 individual studies does not get out of the 95% prediction region around summary point with a negative relationship between sensitivity and specificity, it was judged that heterogeneity between them was not found. Moreover, the negative correlation between sensitivity and specificity on the SROC curve indicated that a higher cut-off value can lead to the increase of sensitivity with some degree of decrease in specificity. As a single measure of diagnostic accuracy, a pooled DOR is 2.20 (95% CI 1.67 ~ 2.91) with non-significant difference (p = 0.30, I2 = 14.7%). AUC is 0.66. The higher DOR and the closer AUC to1, the closer the shape of ROC curve to top left corner, which indicates that diagnostic accuracy is better as much.

**Fig 6:**
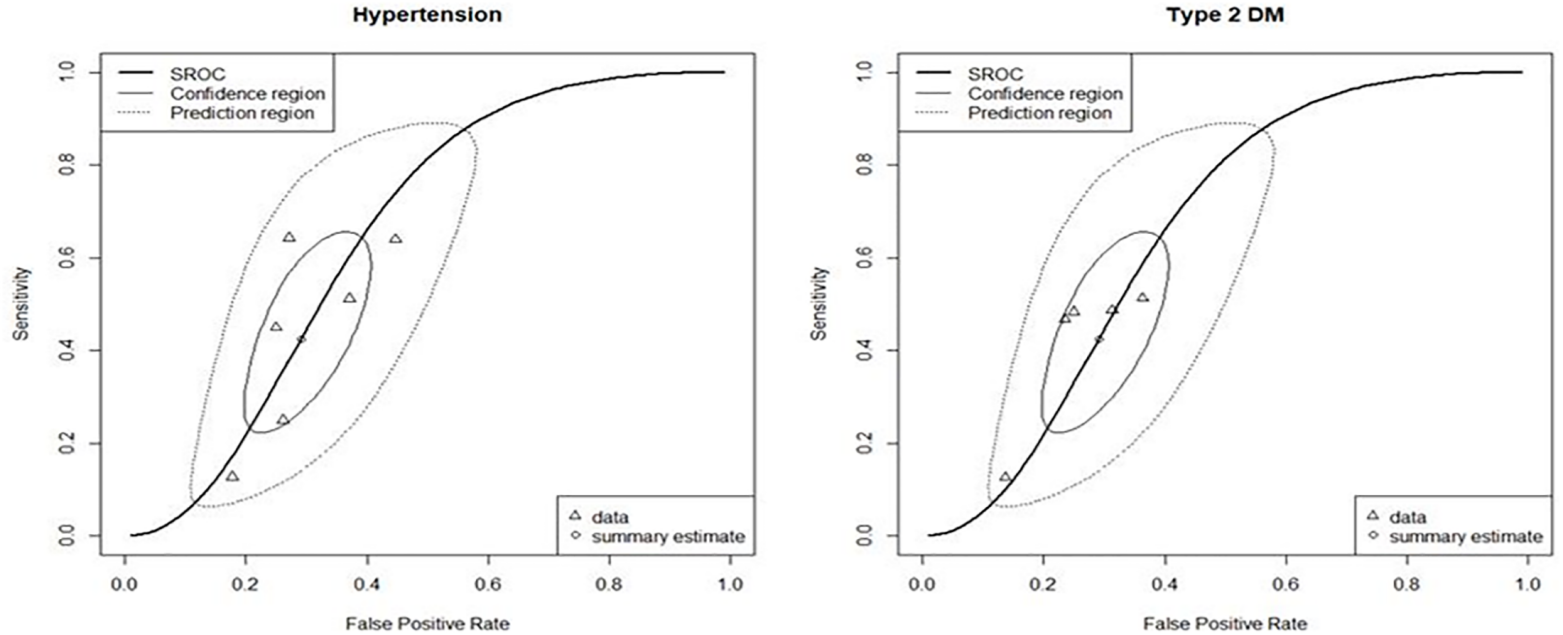
SROC plots of sensitivity and specificity based on cut-off of 6 using bivariate model in the subgroup of hypertension and Type2DM.

As shown in Fig 6, the results of subgroup analysis by disease type are as follows: hypertension group (reference standard: blood control in 5 studies, MPR in 1 study) has sensitivity of 0.43 (95% CI 0.26 ~ 0.61) and specificity of 0.71 (95% CI 0.62 ~ 0.79) with no heterogeneity between them; type2DM group (reference standard: HbA1c in 4 studies, fasting blood glucose in 1 study) has sensitivity 0.40 (95% CI 0.25 ~ 0.57) and specificity 0.74 (95% CI 0.64 ~ 0.82) with no heterogeneity across them. As osteoporosis disease included only one study, the subgroup analysis was not undertaken and it showed the sensitivity of 0.48 (95% CI 0.43 ~ 0.54) and the specificity of 0.82 (95% CI 0.75 ~ 0.88). There seems not to be heterogeneity between hypertension group and type2DM group. The shape of two subgroups’ SROC curves seems to be similar each other as it is shown in Fig 6. In practice, AUC (0.65 vs 0.65) and DORs (2.01 vs 2.04) in hypertension and type2DM group are almost same in values.

#### Other validities

The underlying construct of MMAS-8 from factor analysis was analyzed in 16 out of 28 studies—1 structure in 10 studies, 2 in 2 studies, and 3 in 4 studies. There were 13 studies that conducted factor loading analysis in all items with its loading value equal or over 0.3 in 9 studies, 0.2 in 3 studies and 0.1 in 1 study. Known-group validity was dealt in 13 out of 21 studies to measure correlation between clinical response and the degree of adherence to medication, and all presented significant association between them. Convergent validity was measured in 14 studies, which 1 study utilized 9-item Hill-Bone Medication Compliance Subscale (r = -0.65, P < 0.001), another 1 study with MA-VAS (r = 0.57~0.75, P < 0.01), 6 studies with MMAS-4 (r = 0.77~0.92, P < 0.01), and the remaining 8 studies with different types of medication compliance questionnaires.

### Additional analysis

Among the 10 studies presenting the distribution of the responses to each item of MMAS-8 the items in which respondents greater than 90% selected responses that indicated adherence in majority of them, were as follows; item 3 (Have you stopped taking medicine when worse?), item 5 (Did you take medicine yesterday?), and item 6 (Have you stopped taking medicine when better?) (S7 Appendix). For 12 studies presenting the Cronbach’s if deleted of each item of MMAS-8, the Cronbach’s α if deleted of item 5 was greater than the Cronbach’s α in total in most of them. (S7 Appendix).

### Overall results

The Cronbach's α values varied from 0.31 to 0.83 among 25 studies. According to subgroup analysis of Cronbach’s α, an only type 2 diabetes group (7 studies) and had 0.67 (95% CI 0.65 to 0.69) and 0.77 (95% CI 0.71 to 0.83), respectively, as the pooled estimates of Cronbach’s α. For ICC, 9 studies showed moderate to high values ranging from 0.68–0.91; however, one had a low value (ICC = 0.24) [39]. The pooled estimate of ICC in the diabetes group (3 studies) was high (0.81; 95% confidence interval, 0.75 to 0.85). The pooled estimates of ICCs in the type 2 diabetes group (3 studies) and osteoporosis group (2 studies) were 0.81 (95% CI, 0.75 to 0.85) and 0.84 (95% CI 0.74 to 0.85) by subgroup analysis of ICC. For a cut-off value of 6, the pooled sensitivity and specificity in 12 studies were 0.43 (95% CI, 0.33 to 0.53) and 0.73 (95% CI, 0.68 to 0.78), respectively.

## Discussion

The MMAS-8 had acceptable pooled estimates of internal consistency in type 2 diabetes, osteoporosis, and hypertension (translated version in a female-dominant subgroup) groups. However, only the diabetes group had a pooled estimate without heterogeneity regarding test-retest reliability. Criterion validity of the MMAS-8 from this study is not enough to apply it to a research requiring high levels both in sensitivity and specificity of measurement of medication adherence behavior.

This study did have some limitations. At the study level, nearly half of all included studies dealing with diagnostic accuracy had a reference standard with high risk or unclear description in both the assessment of risk of bias and applicability. It has been reported that Cronbach’s alpha is not appropriate for internal consistency and reliability of a test [40, 41], but all studies included in this review used it. At the outcome level, almost of individual studies calculating sensitivity and specificity used the cut-off value of 6, suggested by Morisky [14]. If they had addressed sensitivity and specificity by different cut-off values, more information of diagnostic accuracy of MMAS-8 could have been provided. This study showed a negative correlation between sensitivity and specificity on the SROC curve indicating that a higher cut-off value can lead to the increase of sensitivity with some degree of decrease in specificity. At the review level, since the number of studies included in this systematic review was relatively smaller comparing to the number of covariates which could be resource for heterogeneity, meta-regression could have not been undertaken to identify the covariates of heterogeneity.

It needs to be noted that considerable room remains for improving reliability and validity. 8 of 10 studies that presented adherent responses in terms of individual items, reported that item 5 (“Did you take medicine yesterday?”) is difficult to discriminate patient behavior due to ‘yes’ response of more than 90% among subjects in each study. This point was also raised in the study by DiBonaventura et al. using Item response theory [42]. If item 5 is replaced with another item sensitive to medication adherence, the modified scale can be better in criterion validity. Next, this study lacks various cut-off levels of MMAS-8 total score. Morisky suggested 6 as a cut-off value of MMAS-8 when developing it, so almost studies in publication showed outcomes regarding criterion validity by the cut-off value of 6. Criterion validity needs to be reassessed by another values like 7 and 8 in the future.

## Supporting information

S1 Appendix PRISMA guideline checklist. (DOCX)

S2 Appendix A protocol. (PPTX)

S3 Appendix Search strategy. (DOCX)

S4 Appendix General characteristics of the included studies. (DOCX)

S5 Appendix Assessment of methodological quality by QUADAS-2. (DOCX)

S6 Appendix True positive (TP), true negative (TN), false positive (FP) and false negative (FN) values sensitivity and specificity (95% CI), diagnostic odds ratio (DOR) of each study using cut-offs of 6. (DOCX)

S7 Appendix Additional analysis (per-item analysis). (DOCX)
