# Correction: Accuracy of a screening tool for medication adherence: A systematic review and meta-analysis of the Morisky Medication Adherence Scale-8

**DOI:** 10.1371/journal.pone.0196138

**Published:** 2018-04-17

**Authors:** Sun Jae Moon, Weon-Young Lee, Jin Seub Hwang, Yeon Pyo Hong, Donald E. Morisky

The authors provide the following update to the Competing Interests statement:

DEM holds a copyright for Morisky Medication Adherence Scale-8 (MMAS-8), is one of its authors, and collects fees in exchange for licenses to use this scale in research. This does not alter the authors’ commitment to objectivity in research and our adherence to PLOS ONE policies on sharing data and materials.
