# Supplementary material for: Accuracy of a screening tool for medication adherence: A systematic review and meta-analysis of the Morisky Medication Adherence Scale-8
Source: PLoS One. 2017 Nov 2;12(11):e0187139. doi: 10.1371/journal.pone.0187139 (PMC5667769; doi:10.1371/journal.pone.0187139)
Supplement: S2 Appendix — (PPTX) [file pone.0187139.s002.pptx]

## Slide 1
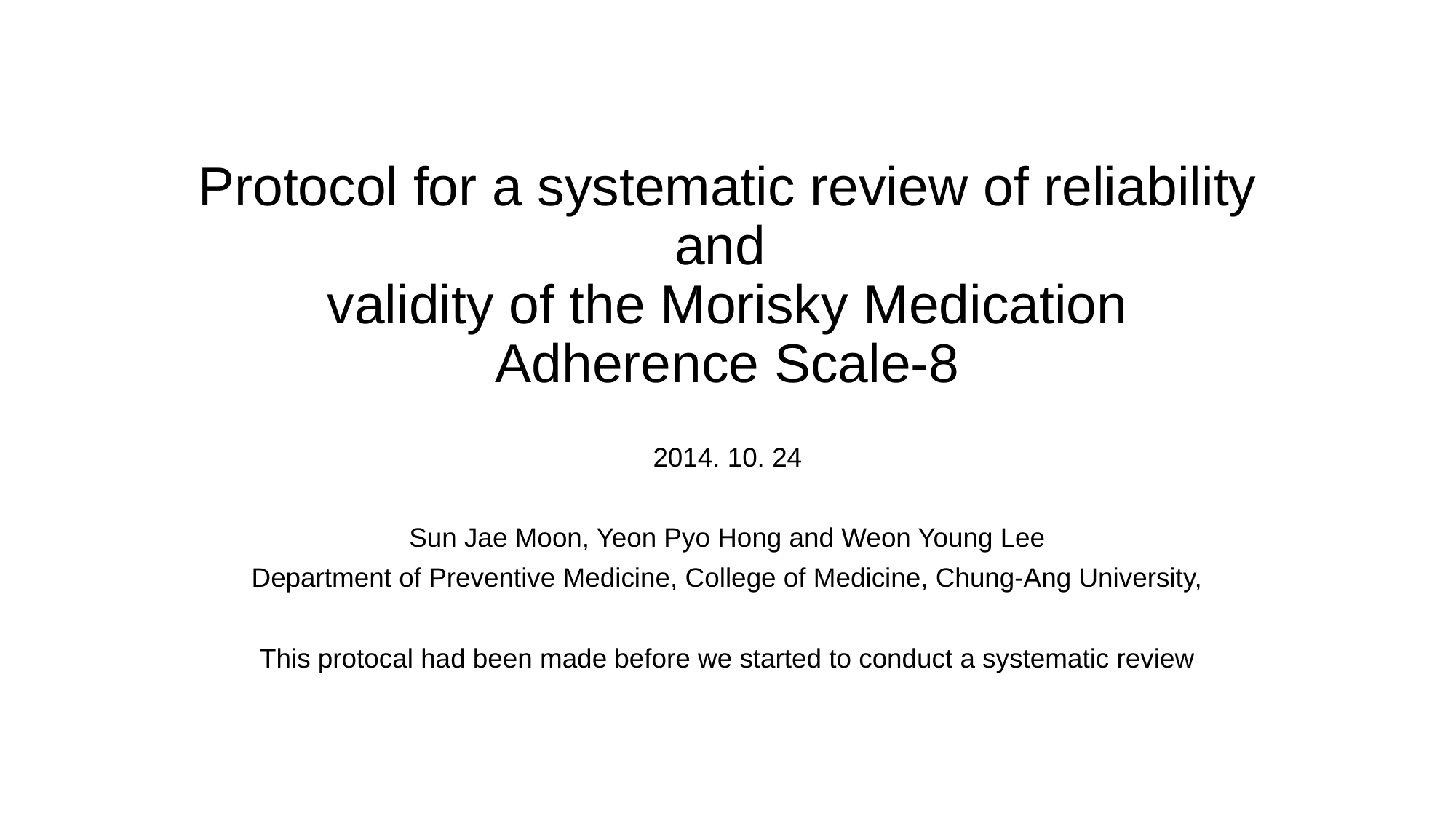

# Protocol for a systematic review of reliability and validity of the Morisky Medication Adherence Scale-8
2014. 10. 24
Sun Jae Moon, Yeon Pyo Hong and Weon Young Lee
Department of Preventive Medicine, College of Medicine, Chung-Ang University,
This protocal had been made before we started to conduct a systematic review

## Slide 2
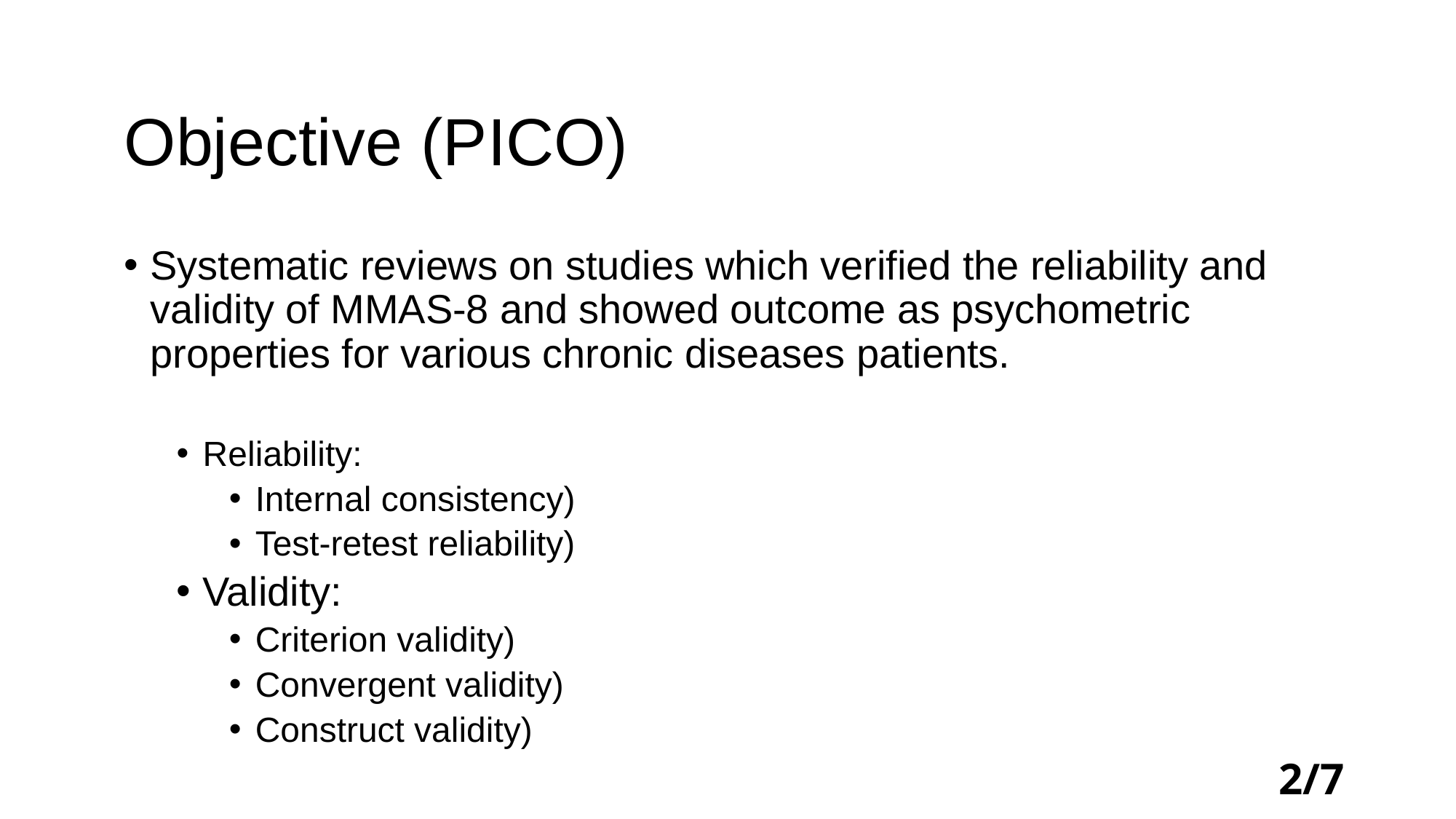

# Objective (PICO)
Systematic reviews on studies which verified the reliability and validity of MMAS-8 and showed outcome as psychometric properties for various chronic diseases patients.
Reliability:
Internal consistency)
Test-retest reliability)
Validity:
Criterion validity)
Convergent validity)
Construct validity)
2/7

## Slide 3
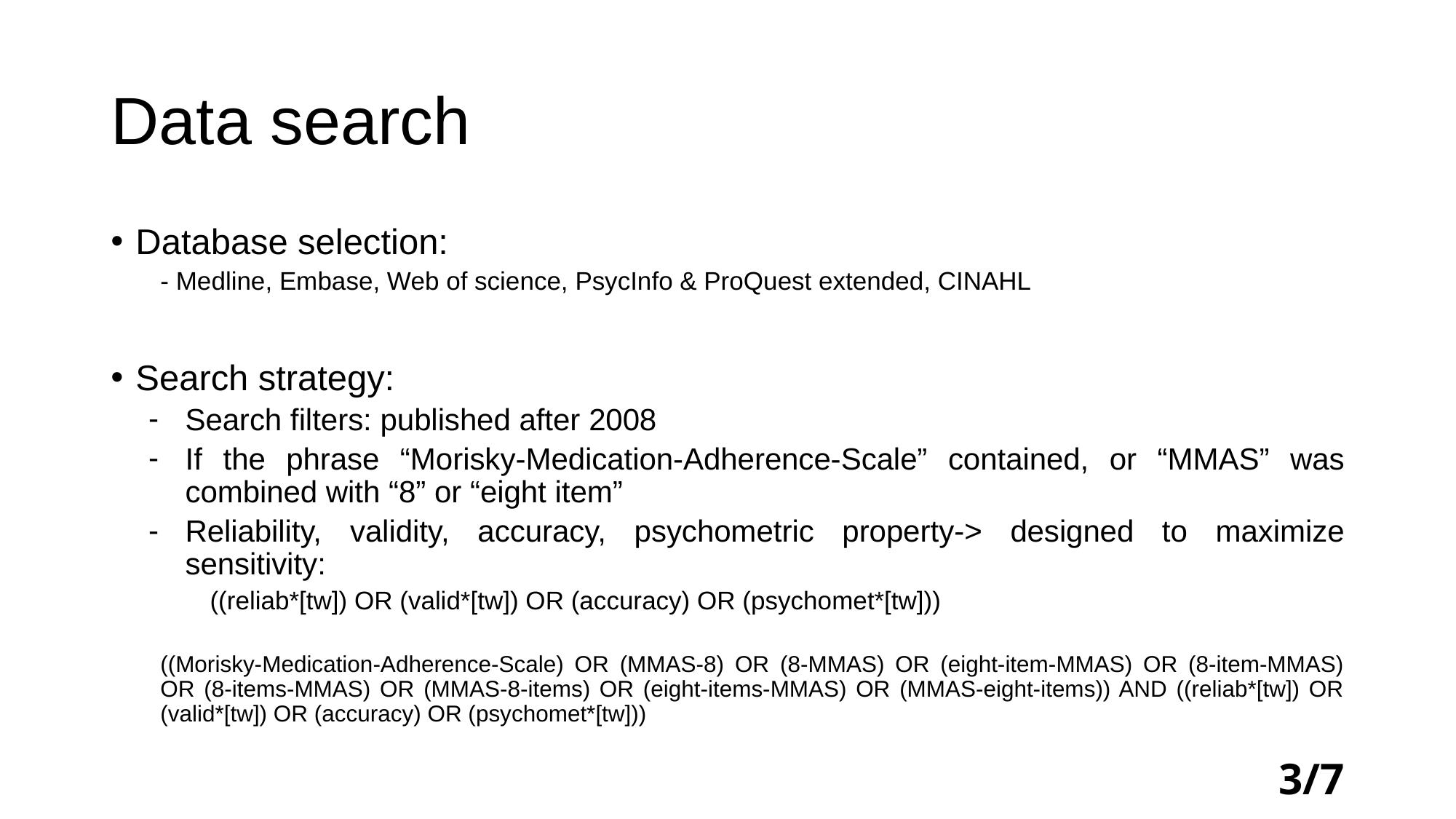

# Data search
Database selection:
- Medline, Embase, Web of science, PsycInfo & ProQuest extended, CINAHL
Search strategy:
Search filters: published after 2008
If the phrase “Morisky-Medication-Adherence-Scale” contained, or “MMAS” was combined with “8” or “eight item”
Reliability, validity, accuracy, psychometric property-> designed to maximize sensitivity:
((reliab*[tw]) OR (valid*[tw]) OR (accuracy) OR (psychomet*[tw]))
((Morisky-Medication-Adherence-Scale) OR (MMAS-8) OR (8-MMAS) OR (eight-item-MMAS) OR (8-item-MMAS) OR (8-items-MMAS) OR (MMAS-8-items) OR (eight-items-MMAS) OR (MMAS-eight-items)) AND ((reliab*[tw]) OR (valid*[tw]) OR (accuracy) OR (psychomet*[tw]))
3/7

## Slide 4
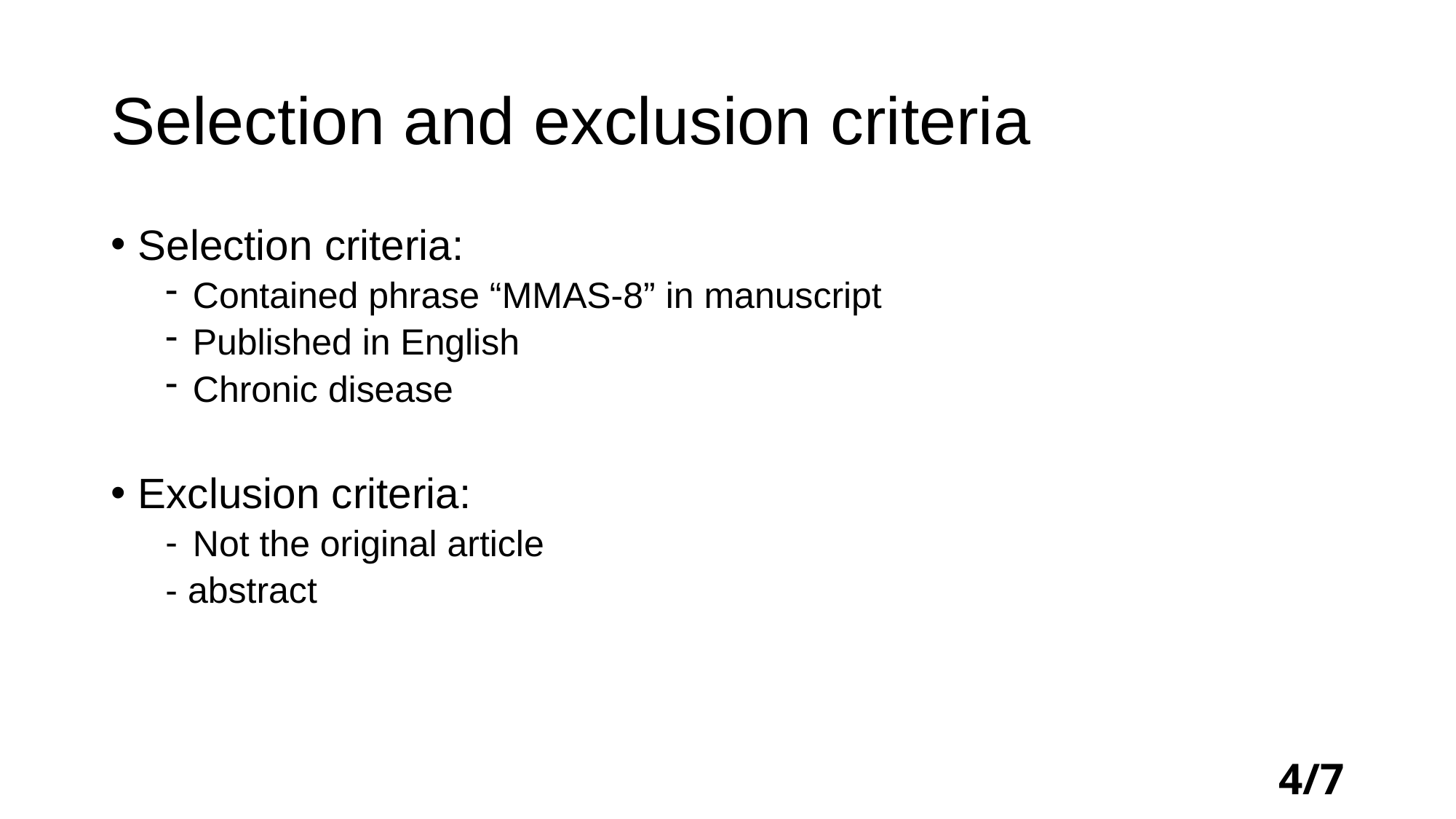

# Selection and exclusion criteria
Selection criteria:
Contained phrase “MMAS-8” in manuscript
Published in English
Chronic disease
Exclusion criteria:
Not the original article
- abstract
4/7

## Slide 5
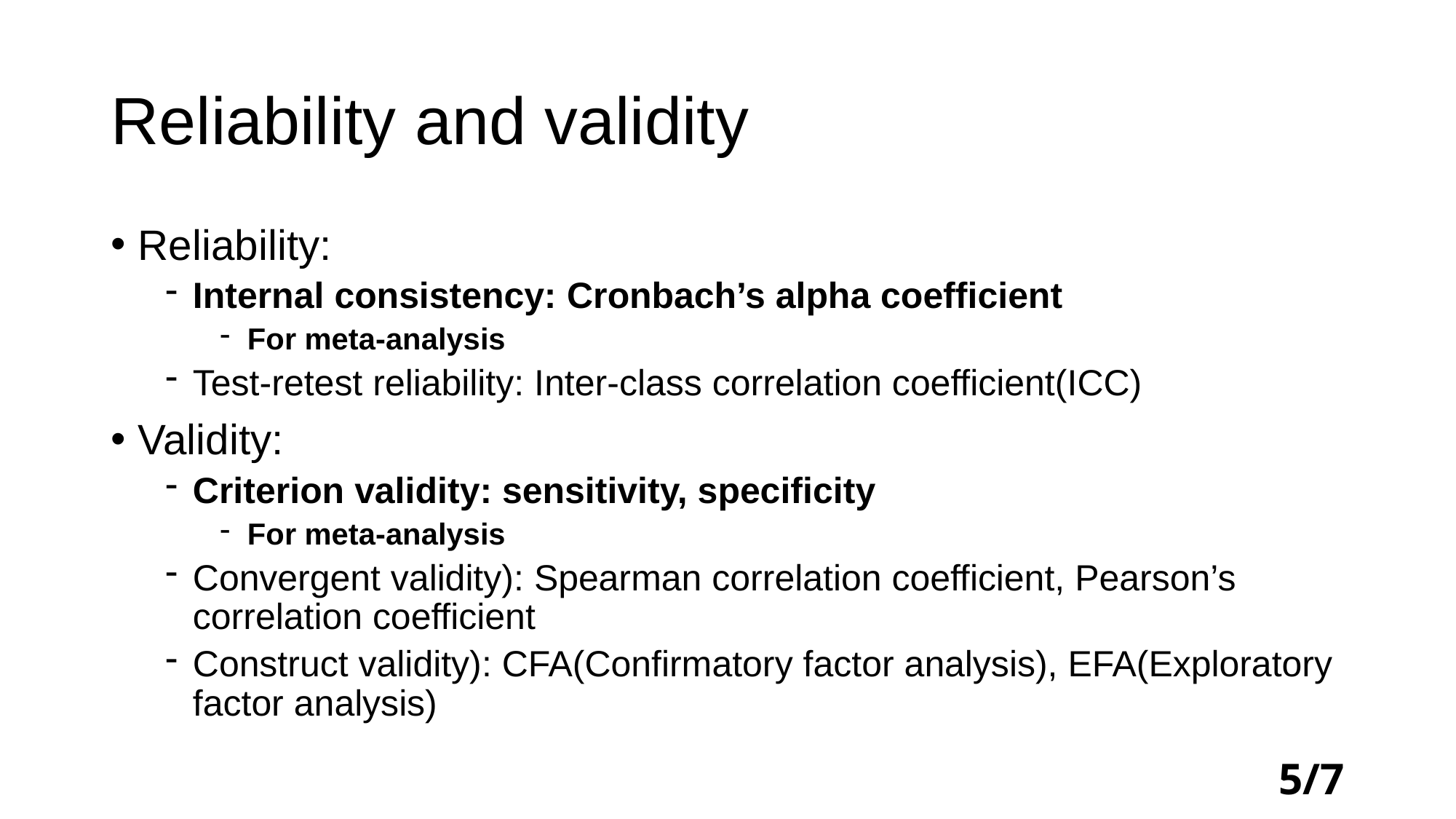

# Reliability and validity
Reliability:
Internal consistency: Cronbach’s alpha coefficient
For meta-analysis
Test-retest reliability: Inter-class correlation coefficient(ICC)
Validity:
Criterion validity: sensitivity, specificity
For meta-analysis
Convergent validity): Spearman correlation coefficient, Pearson’s correlation coefficient
Construct validity): CFA(Confirmatory factor analysis), EFA(Exploratory factor analysis)
5/7

## Slide 6
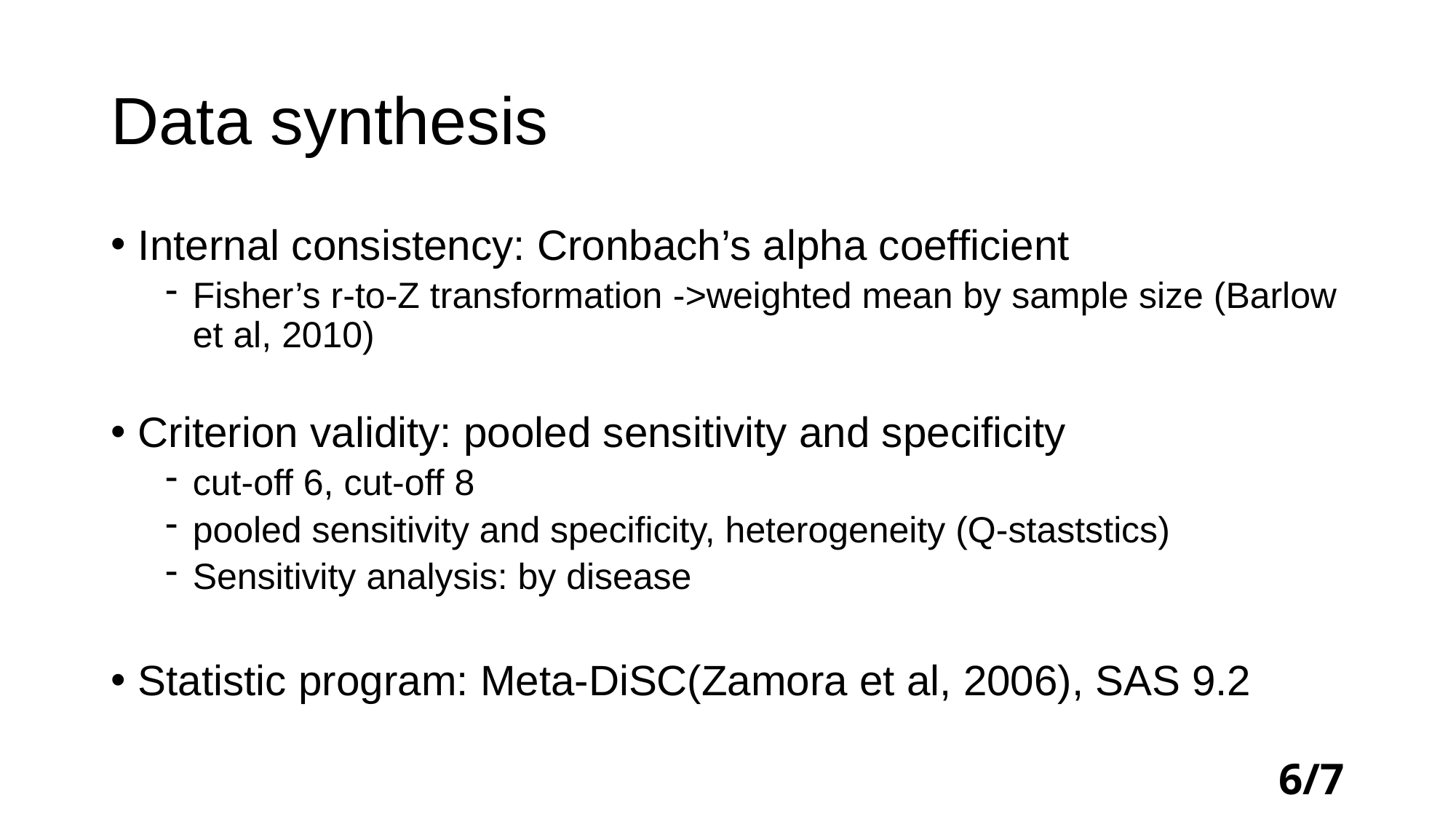

# Data synthesis
Internal consistency: Cronbach’s alpha coefficient
Fisher’s r-to-Z transformation ->weighted mean by sample size (Barlow et al, 2010)
Criterion validity: pooled sensitivity and specificity
cut-off 6, cut-off 8
pooled sensitivity and specificity, heterogeneity (Q-staststics)
Sensitivity analysis: by disease
Statistic program: Meta-DiSC(Zamora et al, 2006), SAS 9.2
6/7

## Slide 7
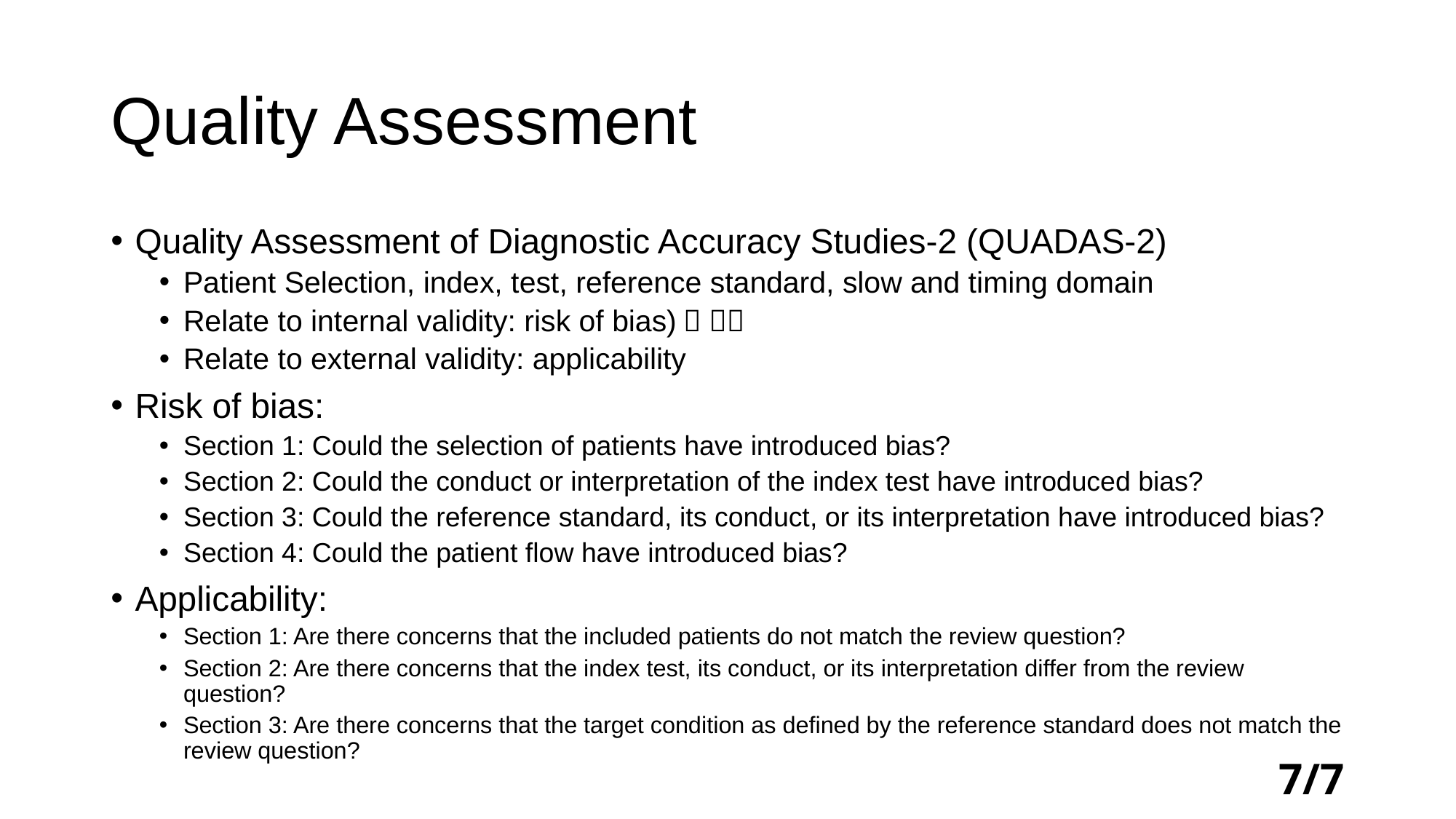

# Quality Assessment
Quality Assessment of Diagnostic Accuracy Studies-2 (QUADAS-2)
Patient Selection, index, test, reference standard, slow and timing domain
Relate to internal validity: risk of bias)을 평가
Relate to external validity: applicability
Risk of bias:
Section 1: Could the selection of patients have introduced bias?
Section 2: Could the conduct or interpretation of the index test have introduced bias?
Section 3: Could the reference standard, its conduct, or its interpretation have introduced bias?
Section 4: Could the patient flow have introduced bias?
Applicability:
Section 1: Are there concerns that the included patients do not match the review question?
Section 2: Are there concerns that the index test, its conduct, or its interpretation differ from the review question?
Section 3: Are there concerns that the target condition as defined by the reference standard does not match the review question?
7/7
