# Supplementary material for: Accuracy of a screening tool for medication adherence: A systematic review and meta-analysis of the Morisky Medication Adherence Scale-8
Source: PLoS One. 2017 Nov 2;12(11):e0187139. doi: 10.1371/journal.pone.0187139 (PMC5667769; doi:10.1371/journal.pone.0187139)
Supplement: S3 Appendix — (DOCX) [file pone.0187139.s003.docx]

S3 Appendix. Search strategy

Database: PubMed

Published Date: 20080101-20151231

| 01. | ("Morisky Medication Adherence Scale 8"[All Fields] OR "8 Morisky Medication Adherence Scale"[All Fields]) | 14 |
| --- | --- | --- |
| 02. | ("eight Morisky Medication Adherence Scale"[All Fields] OR "Morisky Medication Adherence Scale eight"[All Fields]) | 0 |
| 03 | (“MMAS 8”[All Fields] OR “8 MMAS”[All Fields]) | 73 |
| 04 | (“MMAS eight”[All Fields] OR “eight MMAS”[All Fields]) | 0 |
| 05. | 1 OR 2 OR 3 OR 4 | 82 |
| 06. | “MMAS"[All Fields] OR “Morisky-Medication-Adherence-Scale"[All Fields] | 340 |
| 07. | (8-item*) OR (eight-item*) | 1,337 |
| 08. | 6 AND 7 | 98 |
| 09. | 5 OR 8 | 123 |

Database: Embase

Published Date: 20080101-20151231

| 01. | (‘Morisky Medication Adherence Scale 8’ OR ‘8 Morisky Medication Adherence Scale’) | 20 |
| --- | --- | --- |
| 02. | (‘eight Morisky Medication Adherence Scale’ OR ‘Morisky Medication Adherence Scale eight’) | 0 |
| 03 | (‘MMAS 8’ OR ‘8 MMAS’) | 144 |
| 04 | (‘MMAS eight’ OR ‘eight MMAS’) | 0 |
| 05. | 1 OR 2 OR 3 OR 4 | 154 |
| 06. | ‘MMAS’ OR ‘Morisky Medication Adherence Scale’ | 677 |
| 07. | (8-item$) OR (eight-item$) | 1,181 |
| 08. | 6 AND 7 | 166 |
| 09. | 5 OR 8 | 231 |

Database: The Cumulative Index to Nursing and Allied Health Literature (CINAHL)

Published Date: 20080101-20151231

| 01. | (Morisky-Medication-Adherence-Scale-8 OR 8-Morisky-Medication-Adherence-Scale) | 2 |
| --- | --- | --- |
| 02. | (eight-Morisky-Medication-Adherence-Scale OR Morisky Medication-Adherence-Scale-eight) | 2 |
| 03 | (MMAS-8 OR 8-MMAS) | 13 |
| 04 | (MMAS-eight OR eight-MMAS) | 13 |
| 05. | 1 OR 2 OR 3 OR 4 | 14 |
| 06. | (MMAS OR Morisky-Medication-Adherence-Scale) | 231 |
| 07. | (8-item*) OR (eight-item*) | 432 |
| 08. | 6 AND 7 | 27 |
| 09. | 5 OR 8 | 30 |

Database: PsycINFO

Publication date (filter): 2008-2015 (Entered date: 01/ 01/ 2008 – 2015)

| 01. | (Morisky-Medication-Adherence-Scale-8 OR 8-Morisky-Medication-Adherence-Scale) | 14 |
| --- | --- | --- |
| 02. | (eight-Morisky-Medication-Adherence-Scale OR Morisky Medication-Adherence-Scale-eight) | 0 |
| 03 | (MMAS-8 OR 8-MMAS) | 17 |
| 04 | (MMAS-eight OR eight-MMAS) | 0 |
| 05. | 1 OR 2 OR 3 OR 4 | 25 |
| 06. | (MMAS OR Morisky-Medication-Adherence-Scale) | 165 |
| 07. | (8-item*) OR (eight-item*) | 917 |
| 08. | 6 AND 7 | 24 |
| 09. | 5 OR 8 | 34 |
