# Supplementary material for: Accuracy of a screening tool for medication adherence: A systematic review and meta-analysis of the Morisky Medication Adherence Scale-8
Source: PLoS One. 2017 Nov 2;12(11):e0187139. doi: 10.1371/journal.pone.0187139 (PMC5667769; doi:10.1371/journal.pone.0187139)
Supplement: S4 Appendix — (DOCX) [file pone.0187139.s004.docx]

S4 Appendix. General characteristics of the included studies

|  | Publication type | Country | Country and clinical setting | Disease | Sample size | Age, mean years (SD) | Sex (Female, %) | High adherence group % (% of MMAS-8 score ≥6) | Reference standard | Measurement of index test (MMAS-8) | Measurement of reference | Meta-analysis of reliability indices | Meta-analysis of sensitivity and specificity |
| --- | --- | --- | --- | --- | --- | --- | --- | --- | --- | --- | --- | --- | --- |
| Ashur 2015 | Journal article | Libya | Waiting area of the outpatient clinics | Type 2 diabetes mellitus | 103 | 52.7 (8.6) | 68.9 | 53.3 | HbA1c in control^a^ | Asked each subject to complete the questionnaire himself/herself in an unknown setting | Patients were asked to provide their most recent HbA1c level | Cronbach’s α | Cut-off <6 and <8^b^ |
| Tandon 2015 | Journal article | Togo | Convenience sample of National Togolese Diabetes Clinic in Lome´ | Type 2 diabetes mellitus | 154 | 57.5 (10) | 73.0 | 64.2 | Fasting blood glucose level^c^ | Asked subject to complete the questionnaire at the clinic | Unknown setting | Cronbach’s α | Cut-off <6 and <8^b^ |
| Pandey 2015 | Journal article | USA | Hypertension  specialty clinic at the University of Texas Southwestern  Medical Center | Hypertension (treatment resistant)^d^ | 47 | 52.4 | 46.9 | 74.4 | Therapeutic drug monitoring (therapeutic range of drugs) | Asked each subject to complete the questionnaire at the clinic | Serum/plasma assays of therapeutic range of drugs (Compliance with Clinical Laboratory Improvement  Act, CLIA–certified laboratories) | Cronbach’s α | Cut-off <6 and <8^b^ |
| Zongo 2015 | Journal article | Canada  (Quebec) | Diabète Québec (Quebec provincial advocacy association patients with diabetes) membership  file | Type 2 diabetes mellitus | 153 | 64.3 (59.1–68.2; 25th percentile –75th percentile) | 37.9 | 86.9 | HbA1c in control^a^ | Asked each subject to complete the questionnaire on the web | HbA1c level was measured using a self-measuring device (A1C NowSelfCheck , NY, USA); results were sent by phone or e-mail | - | Cut-off <6 and <8^b^ |
| Arnet 2015 | Journal article | Switzer-land | Convenience sample of 19 general practitioners | Thromboembolic disease^e^ | 70 | 65.7 ± 9.9 | 31.4 | 64.3 | - | Face-to-face interview at the clinic by unknown interviewer | Electronic database | Cronbach’s α | - |
| Pareja Martínez 2015 | Journal article | Spain | Pharmacy | Hypertension | 100 | 65.5 (10.8) | 43.0 | 85.0 | Blood pressure in control^f^ | Unknown setting | Electronic sphygmomanometer by the pharmacist | Cronbach’s α | Cut-off <6 and <8^b^ |
| Moharamzad 2014 | Journal article | Iran | Cardiology clinic of university hospital, private cardiology office, pharmacy, and private general practitioner | Hypertension | 200 | 59.7 ±27.2 | 58.0 | 46.0 | Blood pressure in control^f^ | Asked each subject to complete the questionnaire himself/herself in an unknown place | Calibrated mercury sphygmomanometer by the researchers | Cronbach’s α | Cut-off <6 and <8^b^ |
| Hacıhasanoğlu Asilar 2014 | Journal article | Turkey | Family Health Centers (FHC) | Hypertension | 196 | 61.8±11.4 | 60.7 | 41.3 | - | Face-to-face interview at the clinic by unknown interviewer | - | Cronbach’s α | - |
| Ho 2014 | Abstract^g^ | Taiwan | Regional hospital in eastern Taiwan | Hypertension | 604 | Unknown | Unknown | 57.5 | ^-^ | Unknown setting | Unknown setting | Cronbach’s α | - |
| Ronghui 2014 | Abstract^g^ | China | Convenience sample of community patients with hypertension in Beijing | Hypertension | 2908 | 60.62 (9.14) | 61.5 | 74.4 | - | Unknown setting | - | Cronbach’s α | - |
| Reynolds 2014 | Journal article | USA | Kaiser Permanente Southern California (KPSC) | Osteoporosis | 400 | 71.6 (8.6) | 100.0 | 61.5 | Medication possession ratio^h^ | Mailed (postage-paid) survey by unknown interviewer | Electronic health record (EHR) | Cronbach’s α, Inter-class correlation coefficient  (1-3 weeks) | Cut-off <6 and <8^b^ |
| Yan 2014 | Journal article | China | 4 major hospitals with cardiac care unit in Guangzhou | Myocardial infarction | 176 | 64.6 (12.1) | 24.4 | 53.9 | - | Asked each subject to complete the questionnaire himself/herself at the clinic | - | Cronbach’s α, Inter-class correlation coefficient  (4 weeks) | - |
| De Oliveira-Filho 2014 | Journal article | Brazil | 6 family health units of the Unified Health System | Hypertension | 937 | 57.1 (12.7) | 71.5 | 53.3 | Blood pressure in control^f^ | Home interviews by trained pharmaceutical science students | Calibrated mercury sphygmomanometer by trained pharmaceutical science students at home | Cronbach’s α | Cut-off <6 and <8^b^ |
| Yang 2014 | Journal article | China | Huashan Hospital | Seizure | 111 | 32.9 (14.9) | 50.5 | 79.3 | - | Asked each subject to complete the questionnaire himself/herself in an unknown setting | - | Cronbach’s α, Inter-class correlation coefficient  (4 weeks) | - |
| Kim 2014 | Journal article | South Korea | Chung-Ang University Hospital | Hypertension | 373 | 57.2 (11.20) | 45.0 | 67.3 | Blood pressure in control^f^ | Face-to-face interview at the clinic by trained nurse | Calibrated mercury sphygmomanometer by trained nurse at clinic | Cronbach’s α, Inter-class correlation coefficient  (2 weeks) | Cut-off <6 and <8^b^ |
| DiBonaventura2014 | Journal article | USA | 2012 US National Health and Wellness Survey (NHWS) data set | Type 2 diabetes mellitus | 1198 | 60.65 (10.74) | 38.6 | Unknown | ^-^ | Unknown setting | Patients were asked to provide their most recent HbA1c level | Cronbach’s α | - |
| Goodhand 2013 | Journal article | UK | Bart’s and the London NHS Trust (a tertiary adult and pediatric IBD center) | Inflammatory bowel disease | 144 | 30.3 | 56.9 | Unknown | Drug metabolite (6-thioguanine)^i^ | Unknown setting | Purine Laboratory at St. Thomas’ NHS Trust | - | Cut-off <6^b^ |
| Wang 2013 | Journal article | China | Research Center of Jiangsu Province Geriatric Institute | Type 2 diabetes mellitus | 182 | 64.9±9.57 | 72.5 | 73.1 | ^-^ | Asked each subject to complete the questionnaire himself/herself in an unknown setting | Electronic database | Cronbach’s α, Inter-class correlation coefficient  (4 weeks) | - |
| Shin 2013 | Journal article | South Korea | 3 primary health care posts | Hypertension | 92 | 73.24 (6.50) | 79.3 | 66.3 | Blood pressure in control^f^ | Asked each subject to complete the questionnaire himself/herself in an unknown setting | Manually measured by community health practitioners at the clinic | - | Cut-off <6 and <8^b^ |
| Feudjo Tepie 2013 | Abstract^g^ | France | French Longitudinal Patient Database | Osteoporosis | 117 | 73.2 (8.1) | 100.0 | Unknown |  | Asked each subject to complete the questionnaire himself/herself at his/her home | Electronic database | Cronbach’s α, Inter-class correlation coefficient  (12 weeks) | - |
| Lee 2013 | Journal article | South Korea | Chung-Ang University Hospital | Type 2 diabetes mellitus | 317 | 59.3±11.2 | 38.5 | 58.4 | HbA1c in control^a^ | Face-to-face interview at the clinic by unknown interviewer | HbA1c level was measured using a high-pressure liquid chromatography variant II analyzer (BioRad, Hercules, CA, USA) | Cronbach’s α, Inter-class correlation coefficient  (2 weeks) | Cut-off <6 and <8^b^ |
| Wang 2012 | Journal article | Singapore | Singapore General Hospital (SGH) | Thromboembolic disease¶ | 151 | 56.0±14.4 | 47.7 | Unknown | Percentage of INR in the therapeutic range^j^ during the past 2 weeks | Face-to-face interview by unknown interviewer | Electronic database | Cronbach’s α | Cut-off <8 |
| Korb-Savoldelli 2012 | Journal article | France | European Georges Pompidou Hospital | Hypertension | 199 | 55.7±14.6 | 42.7 | 81.4 | - | Unknown setting | - | Cronbach’s α, Inter-class correlation coefficient  (4 weeks) | - |
| Reynolds 2012 | Journal article | USA | Kaiser Permanente Southern California (KPSC) | Osteoporosis | 150 | 70.6(9.1) | 100.0 | 69.3 | - | Mailed (postage-paid) survey by unknown interviewer | - | Cronbach’s α, Inter-class correlation coefficient  (1-3 weeks) | - |
| Al-Qazaz 2010 | Journal article | Malaysia | Penang General Hospital | Type 2 diabetes mellitus | 175 | 60.56±9.16 | 53.7 | 61.7 | HbA1c in control^a^ | Face-to-face interview at the clinic by the pharmacist | Electronic database | Cronbach’s α | Cut-off <6 and <8^b^ |
| Kerisit 2010 | Abstract^g^ | USA | Endocrinology clinic | Type 2 diabetes mellitus | 230 | 60.7±12.3 | 57.0 | 68.3 |  | Unknown setting | Electronic database | Cronbach’s α | - |
| Sakthong 2009 | Journal article | Thailand | General Police Hospital in Bangkok | Type 2 diabetes mellitus | 303 | 61.1±11.4 | 71.0 | 54.1 | HbA1c in control^a^ | Face-to-face interview at the clinic by the pharmacist | Electronic database | Cronbach’s α, Inter-class correlation coefficient  (1-2 weeks) | Cut-off <6 and <8^b^ |
| Morisky 2008 | Journal article | USA | Large teaching hospital of the Community Hypertension Intervention Project (CHIP) | Hypertension | 1367 | 52.5 (12.2) | 59.2 | 67.9 |  | Interview at the clinic by a community health worker | Calibrated mercury sphygmomanometer by a certified nurse at the clinic | Cronbach’s α | - |
| ^a^ HbA1c <7.  ^b^ Sensitivity at a cut-off <6 was defined as [true positive; (poorly controlled with MMAS score<6 group)/[non-adherence group by reference standard;(poorly controlled group)]); specificity was defined as [true negative; (poorly controlled with MMAS score<6 group)/[non-adherence group by reference standard; (poorly controlled group)]). Sensitivity at a cut-off <8 was defined as [true positive; (poorly controlled with MMAS score<8 group)/[non-adherence group by reference standard; (poorly controlled group)]), specificity was defined as [true negative; (poorly controlled with MMAS score<6 group)/[non-adherence group by reference standard; (poorly controlled group)]).  ^c^ Subjects with fasting blood glucose level <130 mg/dL were defined as achieving good control, and subjects with fasting blood glucose level > 130 mg/dL were defined as having poor control.  ^d^ The American Heart Association/Committee of the Council for High Blood Pressure Research defines TRH as: (1) failure to achieve office BP <140/90 mm Hg in patients prescribed three or more antihypertensive medications at optimal doses, including if possible a diuretic, or (2) ability to achieve office BP at goal but requiring four or more antihypertensive medications.  ^e^ Thromboembolic disease that requires a medical prescription such as warfarin, aspirin, or clopidogrel.  ^f^ Adherent (systolic BP < 140 mm Hg; diastolic < 90 mm Hg).  ^g^ Abstract-only article, such as conference proceedings.  ^h^ The medication possession ratio (MPR) was calculated as the number of days the medication was supplied, i.e., the sum of the days between the first dispensing and the last refill in the study period (excluding the supply obtained in the last refill) divided by the total number of elapsed days between the first dispensing and the last refill date within the study period. Non-adherent = MPR<0.80, adherent = MPR≥0.80.  ^I^ 6-thioguanine is a metabolite of mesalazine and azathiopurine.  ^j^ International normalized ratio (INR)=2.0-3.0. | | | | | | | | | | | | | |
