# Supplementary material for: Accuracy of a screening tool for medication adherence: A systematic review and meta-analysis of the Morisky Medication Adherence Scale-8
Source: PLoS One. 2017 Nov 2;12(11):e0187139. doi: 10.1371/journal.pone.0187139 (PMC5667769; doi:10.1371/journal.pone.0187139)
Supplement: S5 Appendix — (DOCX) [file pone.0187139.s005.docx]

|  | Risk of bias (internal validity) | | | | Applicability (external validity) | | |
| --- | --- | --- | --- | --- | --- | --- | --- |
| Domain | Patient selection | Index test | Reference standard | Flow and timing | Patient selection | Index test | Reference standard |
| Ashur (2015) | low | high | low | low | low | low | unclear |
| Tandon (2015) | low | low | high | High | low | low | high |
| Pandey (2015) | high | high | low | unclear | low | low | low |
| Zongo (2015) | low | low | high | low | low | low | low |
| Arnet (2015) | high | unclear | - | low | low | low | - |
| Pareja Martínez (2015) | unclear | unclear | unclear | low | low | low | high |
| Moharamzad (2014) | low | low | unclear | low | low | low | low |
| Hacıhasanoğlu Asilar (2014) | low | low | - | low | low | low | - |
| Ho (2014) | unclear | unclear | - | unclear | unclear | unclear | - |
| Ronghui (2014) | unclear | low | - | unclear | unclear | unclear | - |
| Reynolds (2014) | low | low | unclear | low | low | low | unclear |
| Yan (2014) | low | low | - | low | low | low | - |
| De Oliveira-Filho (2014) | low | unclear | unclear | low | low | low | high |
| Yang (2014) | low | low | - | low | low | low | - |
| Kim (2014) | low | unclear | unclear | low | low | low | low |
| DiBonaventura (2014) | low | high | - | low | low | unclear | - |
| Goodhand (2013) | low | unclear | unclear | low | unclear | unclear | unclear |
| Wang (2013) | low | unclear | - | low | low | low | - |
| Shin (2013) | low | low | unclear | low | low | low | low |
| Feudjo Tepie (2013) | unclear | low | - | unclear | unclear | low | - |
| Lee (2013) | low | low | low | low | low | low | low |
| Wang (2012) | low | low | low | high | low | low | unclear |
| Korb-Savoldelli (2012) | low | low | - | low | low | unclear | - |
| Reynolds (2012) | low | low | - | low | low | low | - |
| Al-Qazaz (2010) | low | low | low | low | low | low | unclear |
| Kerisit (2010) | unclear | low | - | unclear | unclear | unclear | - |
| Sakthong (2009) | low | low | low | low | low | low | unclear |
| Morisky (2008) | low | unclear | - | low | low | low | - |

S5 Appendix. Assessment of methodological quality by QUADAS-2
