# Supplementary material for: Accuracy of a screening tool for medication adherence: A systematic review and meta-analysis of the Morisky Medication Adherence Scale-8
Source: PLoS One. 2017 Nov 2;12(11):e0187139. doi: 10.1371/journal.pone.0187139 (PMC5667769; doi:10.1371/journal.pone.0187139)
Supplement: S6 Appendix — (DOCX) [file pone.0187139.s006.docx]

S6 Appendix. True positive (TP), true negative (TN), false positive (FP) and false negative (FN) values sensitivity and specificity (95% CI), diagnostic odds ratio (DOR) of each study using cut-offs of 6.

| Author, year | TP | FP | FN | TN | Disease | Reference standard | Sensitivity (95% CI) | Specificity (95% CI) | DOR (95% CI) |
| --- | --- | --- | --- | --- | --- | --- | --- | --- | --- |
| Al-Qazaz 2010 | 52 | 15 | 59 | 49 | Type 2 diabetes mellitus | HbA1c in control | 0.47 (0.38-0.56) | 0.77 (0.65-0.85) | 2.88 (1.45-5.73) |
| Ashur 2015 | 55 | 3 | 31 | 14 | Type 2 diabetes mellitus | HbA1c in control | 0.64 (0.53-0.73) | 0.82 (0.59-0.94) | 8.28(2.21-31.07) |
| De Oliveira-Filho 2014 | 300 | 127 | 286 | 216 | Hypertension | Blood pressure in control | 0.51 (0.47-0.55) | 0.63 (0.58-0.68) | 1.78 (1.36-2.34) |
| Goodhand 2013 | 17 | 45 | 1 | 81 | Inflammatory bowel disease | Therapeutic range of drugs | 0.94 (0.74-0.99) | 0.64 (0.56-0.72) | 30.60 (3.94-237.57) |
| Kim 2014 | 36 | 86 | 20 | 231 | Hypertension | Blood pressure in control | 0.64 (0.51-0.76) | 0.73 (0.68-0.77) | 4.84 (2.65-8.81) |
| Lee 2013 | 92 | 40 | 97 | 88 | Type 2 diabetes mellitus | HbA1c in control | 0.49 (0.42-0.56) | 0.69 (0.60-0.76) | 2.09 (1.30-3.34) |
| Moharamzad 2014 | 62 | 46 | 35 | 57 | Hypertension | Blood pressure in control | 0.64 (0.54-0.73) | 0.55 (0.46-0.65) | 2.20 (1.24-3.87) |
| Pandey 2015 | 6 | 6 | 18 | 17 | Hypertension (treatment resistant)d | Therapeutic range of drugs | 0.25 (0.12-0.45) | 0.74 (0.54-0.87) | 0.94 (0.26-7.18) |
| Pareja Martinez 2015 | 7 | 8 | 48 | 37 | Hypertension | Blood pressure in control | 0.13 (0.06-0.24) | 0.82 (0.69-0.91) | 0.67 (0.22-2.03) |
| Reynolds 2014 | 131 | 23 | 140 | 106 | Osteoporosis | Medication possession ratio | 0.48 (0.42-0.54) | 0.82 (0.75-0.88) | 4.31 (2.59-7.18) |
| Sakthong 2009 | 99 | 40 | 94 | 70 | Type 2 diabetes mellitus | HbA1c in control | 0.51 (0.44-0.58) | 0.64 (0.54-0.87) | 1.84 (1.14-2.98) |
| Shin 2013 | 18 | 13 | 22 | 39 | Hypertension | Blood pressure in control | 0.45 (0.31-0.60) | 0.75 (0.62-0.85) | 2.46 (1.01-5.94) |
| Tandon 2015 | 30 | 14 | 32 | 42 | Type 2 diabetes mellitus | Fasting blood glucose level | 0.48 (0.36-0.61) | 0.75 (0.62-0.84) | 2.81 (1.29-6.16) |
| Zongo 2015 | 11 | 9 | 76 | 57 | Type 2 diabetes mellitus | HbA1c in control | 0.13 (0.07-0.21) | 0.86 (0.76-0.93) | 0.92 (0.36-2.36) |
