# Supplementary material for: Accuracy of a screening tool for medication adherence: A systematic review and meta-analysis of the Morisky Medication Adherence Scale-8
Source: PLoS One. 2017 Nov 2;12(11):e0187139. doi: 10.1371/journal.pone.0187139 (PMC5667769; doi:10.1371/journal.pone.0187139)
Supplement: S7 Appendix — (DOCX) [file pone.0187139.s007.docx]

S7 Appendix. Additional analysis (per-item analysis)

| Response to be considered adherent | MMAS-8 item number  (1-8)* | Pandey 2015 | Arnet 2015 | Pareja Martínez 2015 | Moharamzad 2014 | Yang 2014 | Kim 2014 | Shin 2013 | Lee 2013 | Wang 2012 | Korb-Savoldelli 2012 |
| --- | --- | --- | --- | --- | --- | --- | --- | --- | --- | --- | --- |
| No response (%) | Item 1 | 66.0% | 87.1% | 87.0% | 59.5% | 46.8% | 63.8% | 70.7% | 53.0% | 55.6% | 68.3% |
| No response (%) | Item 2 | 53.2% | 91.4% | 80.0% | 82.0% | 77.5% | 64.9% | 78.2% | 58.7% | 90.1% | 82.9% |
| No response (%) | Item 3 | 83.0% | 91.4% | 87.0% | 70.0% | 96.4% | 96.5% | 84.8% | 92.7% | 96.0% | 88.9% |
| No response (%) | Item 4 | 87.2% | 95.7% | 89.0% | 62.5% | 87.4% | 81.8% | 71.7% | 80.8% | 94.0% | 87.9% |
| Yes response (%) | Item 5 | 93.6% | 98.6% | 90.0% | 87.0% | 98.2% | 92.2% | 88.0% | 91.8% | 98.7% | 96.0% |
| No response (%) | Item 6 | 91.5% | 97.1% | 95.0% | 62.5% | 96.4% | 92.8% | 88.0% | 94.0% | 97.4% | 96.0% |
| No response (%) | Item 7 | 80.9% | 95.7% | 92.0% | 64.0% | 70.3% | 81.0% | 71.7% | 76.7% | 69.5% | 84.4% |
| No response (%)† | Item 8 | 68.1% | 88.6% | 73.0% | 24.0% | 81.0% | 59.0% | 65.2% | 45.7% | 66.2% | 75.9% |
| - | Sample size | 47 | 70 | 100 | 200 | 111 | 373 | 92 | 317 | 151 | 199 |

| - | MMAS-8 item number  (1-8)* | Ashur 2015 | Tandon 2015 | Arnet 2015 | Moharamzad 2014 | Hacıhasanoğlu Asilar 2014 | Reynolds 2014 | Yan 2014 | De Oliveira-Filho 2014 | DiBonaventura 2014 | Lee 2013 | Reynolds 2012 | Al-Qazaz 2010 |
| --- | --- | --- | --- | --- | --- | --- | --- | --- | --- | --- | --- | --- | --- |
| Cronbach's alpha (Ca) if deleted | Item 1 deleted | - | 0.356 | 0.401 | 0.662 | 0.76 | 0.7 | 0.71 | 0.555 | 0.612 | 0.572 | 0.8 | 0.639 |
|  | Item 2 deleted | - | 0.370 | 0.161 | 0.693 | 0.76 | 0.7 | 0.72 | 0.578 | 0.607 | 0.607 | 0.77 | 0.637 |
|  | Item 3 deleted | - | 0.447 | 0.282 | 0.660 | 0.76 | 0.73 | 0.75 | 0.632 | 0.662 | 0.653 | 0.81 | 0.646 |
|  | Item 4 deleted | - | 0.442 | 0.309 | 0.670 | 0.76 | 0.71 | 0.71 | 0.607 | 0.639 | 0.621 | 0.79 | 0.653 |
|  | Item 5 deleted | 0.71 | 0.449 | 0.328 | 0.685 | 0.8 | 0.73 | 0.79 | 0.617 | 0.701 | 0.649 | 0.79 | 0.667 |
|  | Item 6 deleted | - | 0.466 | 0.317 | 0.660 | 0.76 | 0.72 | 0.75 | 0.599 | 0.667 | 0.643 | 0.79 | 0.621 |
|  | Item 7 deleted | - | 0.513 | 0.219 | 0.687 | 0.79 | 0.72 | 0.76 | 0.645 | 0.648 | 0.65 | 0.81 | 0.657 |
|  | Item 8 deleted | - | 0.424 | 0.202 | 0.599 | 0.76 | 0.68 | 0.73 | 0.623 | 0.614 | 0.607 | 0.7 | 0.641 |
|  | Cronbach's alpha(Ca) | 0.70 | 0.470 | 0.41 | 0.697 | 0.79 | 0.74 | 0.77 | 0.682 | 0.68 | 0.66 | 0.82 | 0.675 |

* For details about the questionnaire items, see [14].

†‘Never’ response to item 8 was recorded as a ‘Yes’ response option.
